# Shelter Distribution and Type Affect Space Use of a Desert Reptile

**DOI:** 10.1002/ece3.70858

**Published:** 2025-02-02

**Authors:** Roy C. Averill‐Murray, J. Daren Riedle

**Affiliations:** ^1^ Arizona Game and Fish Department Phoenix Arizona USA

## Abstract

Animal home ranges derive from the distribution of resources across the landscape. For example, home ranges of most tortoise species in the United States revolve around networks of burrows. However, human disturbances that damage shelters can decrease habitat suitability, individual survival, and population persistence. We investigated effects of burrow distribution and availability on space use of the Sonoran Desert Tortoise *Gopherus morafkai* at two populations with different habitat structures to determine the extent to which habitat capacity is defined by factors subject to management, such as vegetation, relative to more permanent features such as rock shelters. We also demonstrated the superiority of autocorrelated kernel density estimation, illustrating flawed conclusions that could arise from the use of traditional home‐range estimators. Home‐range size increased with the number of available burrows at both sites. At the Florence Military Reservation (FMR), with numerous caliche caves and few rock burrows, larger home ranges effectively compensated for one third the burrow density as that of Sugarloaf Mountain, which predominantly featured rock burrows. Female tortoises had smaller home ranges than males despite having similar burrow densities. Females revisited individual burrows more often than males at Sugarloaf, which may relate to female use of preferred nesting sites; however, lower availability led males to revisit burrows at similar rates as females at FMR. Pairs of tortoises at FMR shared 72% more burrows than pairs at Sugarloaf, and pairs of females shared 33% fewer burrows than female–male pairs across both sites. Space and burrow use at FMR and Sugarloaf are consistent with predictions of how animals choose patches for their home ranges in ways that are optimal with respect to spatially distributed resources. Populations largely reliant on pallets or soil burrows may be more subject to declines due to anthropogenic impacts from grazing or off‐highway vehicle use or due to increasing temperatures.

## Graphical abstract

Animal home ranges derive from the distribution of resources across the landscape. We investigated effects of burrow distribution and availability on space use of the Sonoran Desert Tortoise ( *Gopherus morafkai* ) at two populations with different habitat structures to determine the extent to which habitat capacity is defined by factors subject to management, such as vegetation, relative to more permanent features such as rock shelters. Space and burrow use by *G. morafkai* are consistent with predictions of how animals choose patches for their home ranges in ways that are optimal with respect to spatially distributed resources.

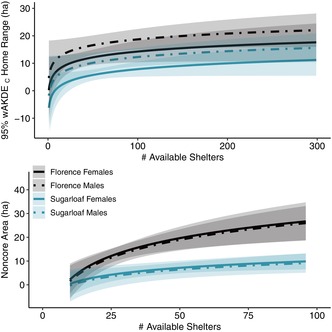

## 1 Introduction

Animal home ranges are shaped by the interaction of individual movements and the distribution of resources necessary for survival and reproduction (Börger, Dalziel, and Fryxell 2008). From an optimal foraging perspective, home‐range size in a landscape with patchy resources should be inversely related to maximum resource density and renewal rate, and core‐area size should decrease as resource density increases, among other predictions (Ford 1983). Subsequent models found that the distribution of resources on the landscape was one of the main determinants of home‐range configuration (Mitchell and Powell 2004, 2012). When resource abundance is high, spatial requirements should be relatively small, but low resource abundance forces animals to expand their home ranges (McMaster and Downs 2009; Castellón, Rothermel, and Bauder 2018; Bista et al. 2022), a relationship that ultimately must be nonlinear to avoid negative or excessively small home ranges (Herfindal et al. 2005; Simcharoen et al. 2014; Doherty, Fist, and Driscoll 2019; Wagler et al. 2024). This model is particularly relevant in regions where resource variability does not change noticeably spatiotemporally, such as equatorial rain forests or highly homogeneous deserts (Mezzini et al. 2023). In addition, limiting resources are often patchy, leading animals to focus use around core areas containing important resources (Samuel, Pierce, and Garton 1985); for example, core‐area size increased with the number of burrows used by Mojave Desert Tortoises *Gopherus agassizii* (Harless et al. 2009).

The connection between abundance and distribution of resources and home‐range size leads to the question of how animals find and use the resources within their home ranges. Many animals remember previously visited sites and tend to revisit them periodically, so memory effects provide a plausible biological mechanism leading to stationary home ranges (Börger, Dalziel, and Fryxell 2008; Van Moorter et al. 2009). More specifically, animal movements are decisions influenced by their internal state, sensory inputs, and past experiences, shaping their home range through a cognitive map that reflects their understanding of the environment (Powell and Mitchell 2012; Fronhofer, Hovestadt, and Poethke 2013; McKeown, Walton, and Willebrand 2020; Krochmal and Roth 2023). In addition to navigation across heterogeneous landscapes, memory can facilitate informed choices about safe shelter locations, den sites, or proven foraging sites (Berger‐Tal and Bar‐David 2015; Seidel and Boyce 2015; Hays et al. 2024). Knowledge of recursive movement patterns with repeat visits to important locations therefore can lead to important insight regarding life history and ecology of populations (Bracis, Bildstein, and Mueller 2018).

Knowledge of a species' space use can help identify strategic spatial units for conservation and can inform targeted management and conservation measures within those areas (Renet et al. 2022; Blake et al. 2023; Delay, Urquhart, and Litzgus 2024; Forrest, Rodríguez‐Recio, and Seddon 2024; Hays et al. 2024). Burrows comprise an important resource for most members of the North American tortoise genus *Gopherus* (Berish and Medica 2014), and burrow distribution within home ranges likely influences the space use of individual tortoises. For example, home ranges of both Sonoran Desert Tortoises *G. morafkai* and Mojave Desert Tortoises *G. agassizii* consist of burrow networks within which individuals spend time near one or more particular burrows before moving to another area (O'Connor et al. 1994; Duda, Krzysik, and Freilich 1999; Riedle et al. 2008; Sullivan et al. 2016). Burrow availability for these two species is crucial, given that tortoises spend approximately 98% of their life inactive in them (Nagy and Medica 1986). They provide nest sites, protection from predators, and refuge from extreme temperatures (Burge 1978; Bulova 1994; Zimmerman et al. 1994; Bailey, Schwalbe, and Lowe 1995; Duda, Krzysik, and Freilich 1999; Mack et al. 2015; Averill‐Murray, Christopher, and Henen 2018). Tortoise density is positively correlated with shelter site density both in the Sonoran Desert (Averill‐Murray, Woodman, and Howland 2002) and in the Mojave Desert (Duda, Kyzysik, and Meloche 2002; Krzysik 2002). Individual tortoises use multiple burrows each season, but they frequently reuse preferred burrows (Woodbury and Hardy 1948; Sullivan et al. 2016), and multiple individuals also may share burrows at the same or at different times (Woodbury and Hardy 1948; Burge 1978; Murray and Klug 1996; Harless et al. 2009). For example, late summer and fall cohabitation of burrows by male and female *G. agassizii* coincides with peaks in male testosterone levels when courtship and mating occur (Rostal et al. 1994; Lance and Rostal 2002).

As a result of the strong links between desert tortoises and their burrows, human disturbances that damage shelter sites (e.g., military training, off‐highway vehicle use or other recreational activity, cattle grazing) will reduce habitat suitability and may impact individual survival and population persistence (Krzysik 1997; Berry, Bailey, and Anderson 2006; Grandmaison, Ingraldi, and Peck 2010). Here, we investigate the effects of burrow distribution and availability on the space use of *G. morafkai* at two populations with markedly different habitat structures. Burrows most often used by *G. morafkai* are associated with relatively permanent rock structures on steep slopes (Averill‐Murray, Allison, and Smith 2014), which is the case at one of our studied populations, while tortoises at the second population predominately occur in less rocky habitat on shallower slopes. Additional study of *G. morafkai* home ranges and burrow use will help parse the extent to which habitat capacity is defined by characteristics subject to management, such as vegetation, in comparison to relatively permanent habitat features such as rock shelters (Averill‐Murray, Fleming, and Riedle 2020).

Finally, comparisons of home ranges across populations and species are often confounded by individual‐level biases, which vary among groups and can propagate into broader population‐level biases if unaddressed (Crane et al. 2021; Fleming et al. 2022). In fact, uncertainties in home‐range estimates historically have not been quantified at all, let alone leveraged in downstream analyses (Fleming et al. 2022). We built on the work of Noonan et al. (2019) and Averill‐Murray, Fleming, and Riedle (2020) to demonstrate the superiority of movement‐based, autocorrelated kernel density estimation (Fleming and Calabrese 2016) to archaic, more familiar methods and to further illustrate implications of failing to account for the biases inherent to those methods.

## 2 Materials and Methods

### 2.1 Study Area

**FIGURE 1:**
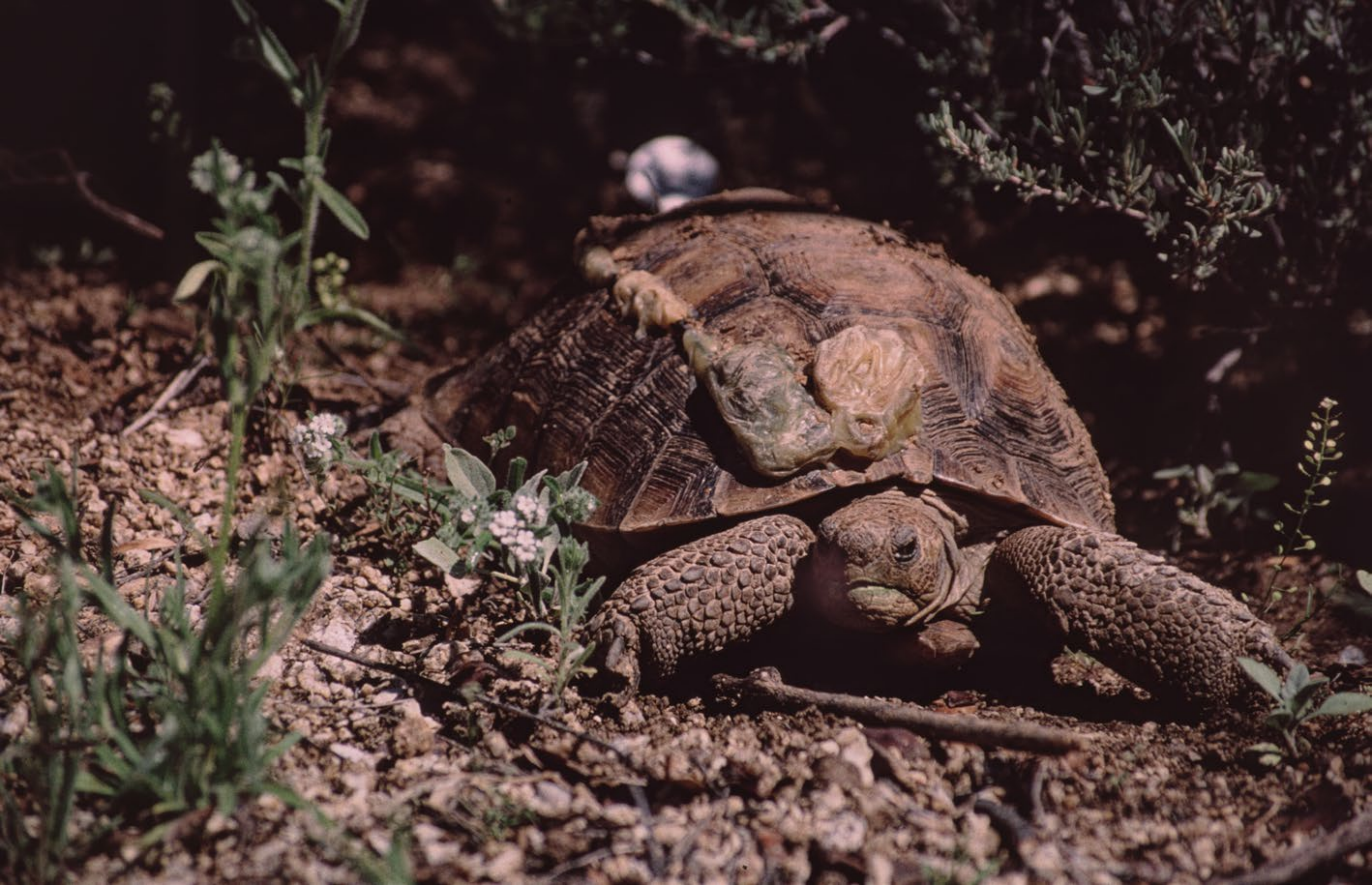
Sonoran Desert Tortoise *Gopherus morafkai* at Sugarloaf Mountain, Arizona. Photo by Roy C. Averill‐Murray.

We studied *G. morafkai* (Figure 1) at two sites in the northeastern Sonoran Desert of Arizona, USA. The Sugarloaf Mountain site was northeast of metropolitan Phoenix in the Tonto National Forest, Maricopa County. The area occurred within the paloverde‐mixed cacti series of the Arizona Upland Subdivision of the Sonoran Desert (Turner and Brown 1982). Arroyos divided a rolling topography of steep, rocky slopes with boulders up to 4‐m diameter and elevations from 549 to 853 m. Sugarloaf generally experienced drought conditions during the period of this study (2000–2003; see below), with a mean standardized precipitation‐evapotranspiration index (SPEI) of −0.48 (0.103 SD; Beguería et al. 2023). SPEI is a standardized index of precipitation that indicates the amount of water surplus or deficit relative to atmospheric demand, with negative values indicating drought conditions (Vicente‐Serrano, Beguería, and López‐Moreno 2010).

The Florence Military Reservation (FMR) site was southeast of Phoenix in Pinal County, approximately 60 km south of Sugarloaf. The landscape at FMR included components of both the Arizona Upland and Lower Colorado River Valley subdivisions of the Sonoran Desert (Brown 1994). Geomorphology was characterized by gently sloping to flat alluvial fans in the north, predominantly filled in with a mix of unconsolidated to weakly consolidated silts, sands, clay, and gravel from the Mineral and Tortilla mountains to the east. The alluvial fans on the eastern portion of the site were bisected by deeply incised washes with exposed caliche layers that are formed when calcium carbonate cements materials such as gravel, sand, clay, and silt into a hardened layer. Caliche layers contained naturally eroded caves and crevices that were often modified by burrowing animals, including tortoises. Our primary study area lay between 543 m and 655 m elevation and included numerous incised washes and a single 10.9‐ha volcanic hill. FMR was also affected by drought during the study, with a mean SPEI of −0.43 (0.130 SD; Beguería et al. 2023).

### 2.2 Data Collection

Animals were handled under the authority and protocols of the Arizona Game and Fish Department and were consistent with Beaupre (2004). At Sugarloaf, we monitored 19 female tortoises (range: 184–289 mm midline carapace length [CL]) weekly using radio telemetry in 1991–1993 and 1997–2005 as part of a reproductive ecology study (Averill‐Murray 2002; Averill‐Murray, Christopher, and Henen 2018). Radio transmitters (< 5% body weight; AVM Instrument Company, Telonics, or Wildlife Materials) were attached to the anterior carapace with 5‐min gel epoxy (Devcon) similar to the method of Boarman et al. (1998; Figure 1). We also monitored a sample of five males (191–265 mm CL). We measured straight midline CL with pottery calipers and a metal tape measure. During the 11 years of radio‐tracking, all burrows used by both telemetered and opportunistically encountered tortoises were marked with numbered aluminum tags (*n* = 522). We use the term “burrow” specifically to refer to a subsurface cavity > 1/2 the tortoise's length either formed by erosion or excavated by a tortoise or another animal (Burge 1978). We categorized burrow types as “rock” (any cover that was provided by rocks greater than the size of the tortoise, including large boulder piles in which the tortoise could not be visualized), “soil” (cover provided by soil or vegetation with no substantial rock component), and “packrat” (freestanding white‐throated woodrat *Neotomoa albigula* nest independent of other shelter types; packrat nests inside rock shelters were categorized as “rock”). We did not individually mark “pallets” (shallow, scraped out areas < 1/2 tortoise length) or other temporary shelters unmodified by the tortoise (e.g., under trees, shrubs, or rock overhangs). We use the term “shelter” generally to refer to any cover used by a tortoise, including burrows, pallets, or other cover.

At FMR, as part of a habitat‐use study, we attached radio transmitters (ATS, AVM, Telonics, or Wildlife Materials) to the anterior carapace of adult tortoises as above (7 males, 11 females; Riedle et al. 2008). During the winter months (November through February) when tortoises were inactive, we located tortoises once a week. During the activity season (March through October), we located tortoises 2–3 times weekly, obtaining both morning and evening locations. From 2000 to 2003, we searched areas in which tortoises might occur within FMR, concentrating on sites suitable for burrow excavation, especially including incised washes with caliche caves and the volcanic hill. We also searched all washes within the study area, whether incised or not, and spent considerable time on the alluvial fans. We marked burrows in which we observed tortoises with individually numbered aluminum tags (*n* = 124), and we mapped locations of all caliche caves large enough to shelter a tortoise ≥ 180 mm CL (*n* = 463). Burrow types at FMR included caliche, rock, soil, and packrat.

At both populations, we used the *spatstat* package in R 4.4.0 to estimate the degree of clustering of burrows across the landscape with an edge‐corrected Clark‐Evans index (*R*) (Baddeley, Rubak, and Turner 2015; R Core Team 2024). *R* is the ratio of the observed mean nearest‐neighbor distance among burrows to that expected for a spatially random Poisson point process of the same intensity; values of *R* > 1 suggest regularity, while *R* < 1 suggests clustering. We estimated the standard deviation of *R* according to Petrere (1985). We also used *spatstat* to illustrate the nearest‐neighbor distance distribution function (*G*) for each population. This function represents the cumulative distribution of distances (*r*) from a typical random burrow to the nearest burrow within the population (Baddeley, Rubak, and Turner 2015). For both *R* and *G*_*r*_, we divided FMR into north and south subpopulations that had access to the rocky hill or not.

### 2.3 Data Analysis

#### 2.3.1 Home Ranges

To directly compare patterns of space and burrow use between sites, we estimated home ranges during the common study period of 2000–2003. Using the same years for both sites reduced potential variation from interannual environmental changes, especially given that annual mean SPEI from 2000 to 2003 was strongly correlated between FMR and Sugarloaf (*r* = 0.98). The lack of effect of drought conditions on space use during the full study at Sugarloaf (Averill‐Murray, Fleming, and Riedle 2020) further justified not incorporating weather‐related covariates in the analyses. We only included adult tortoises (all ≥ 220 mm CL) in the analysis, and we excluded observations between the first and last dates of hibernation each year, estimating the date that each tortoise terminated hibernation as the last day the tortoise was observed inside or < 10 m from its hibernaculum. Following Silva et al. (2022), we used the *ctmm* package in R to calculate variograms, fit movement models, and estimate home ranges with area‐corrected, optimally weighted, autocorrelated kernel density estimation (wAKDE_C_), thereby correcting for bias stemming from autocorrelation in the tracking data (Calabrese, Fleming, and Gurarie 2016; Fleming and Calabrese 2016, 2023). For each tortoise, we plotted the estimated semi‐variance as a function of time lag to visually inspect the autocorrelation structure of the location data; variograms of range residents reveal an asymptote on a timescale that roughly corresponds to the home‐range crossing time, while the plotted semi‐variance of non‐range residents does not approach an asymptote (Fleming et al. 2014; Calabrese, Fleming, and Gurarie 2016). We estimated total home ranges across the entire study period using perturbative Hybrid REML (pHREML) wAKDE_C_ (hereafter, AKDE) conditional on the underlying movement model selected for each tortoise through the model‐selection process within *ctmm* (Fleming et al. 2015; Fleming and Calabrese 2016). In addition to absolute sample sizes (number of relocations) for each tortoise, we report estimated effective samples sizes ($\hat{N}$_area_); $\hat{N}$_area_ is the number of statistically independent locations determined by the duration of the observation period and the timescale over which autocorrelation in position decays (Calabrese, Fleming, and Gurarie 2016; Fleming et al. 2019). Movement models included (1) the independent identically distributed (IID) process, which assumes uncorrelated positions and velocities and is implicit in home‐range estimation with minimum convex polygon (MCP) or conventional kernel density estimation (KDE); (2) the Ornstein–Uhlenbeck (OU) process, which combines Brownian motion (regular diffusion with uncorrelated velocities) with a tendency to remain in a particular area, which thereby applies to data lacking evidence of directional persistence but where restricted space use is apparent; and (3) the Ornstein–Uhlenbeck Foraging (OUF) process that features directional persistence, as indicated by correlated velocities, as well as restricted space use (Calabrese, Fleming, and Gurarie 2016). Finally, the OUf model is a special case of OUF where the autocorrelation timescales for position and velocity cannot be distinguished and is usually only relevant for short tracks of data (“Variograms and Model Selection” vignette in Fleming and Calabrese 2023). We used the “meta” function in *ctmm* to calculate population‐level home range and core area (see below) estimates that account for estimation uncertainty, as well as overall effect sizes between sites and sex‐specific effect sizes within and between sites (Fleming et al. 2022).

We investigated space use in more detail with several measures. For each home range, we estimated core areas as the area encompassed by the 50% AKDE isopleth. We also estimated the proportion of the total (95%) home‐range area contained by the 50% core area (PA) and the intensity of core area use (ICU = core area isopleth/[50% core area/95% AKDE area]; Samuel, Pierce, and Garton 1985). We used the “overlap” function in *ctmm* to calculate the bias‐corrected, Bhattacharyya coefficient overlap of AKDE home ranges between individuals (Winner et al. 2018). We calculated the number of burrows available to each tortoise and estimated burrow densities by clipping vectors of the mapped burrows to the 50% AKDE core areas, the 95% AKDE home ranges, and the 50%–95% noncore areas (i.e., the area within the home range occurring outside the core area) in QGIS 3.34.1. We also counted the number of burrows available within the common portion of each overlapping pair of 95% AKDE home ranges.

We conducted two initial analyses to compare home ranges estimated in this study with (1) MCP estimates for the same individuals for up to 4 years at FMR reported by Riedle et al. (2008) and (2) AKDE estimates based on up to 10 years of data for the same individuals at Sugarloaf reported by Averill‐Murray, Fleming, and Riedle (2020). To compare MCP and AKDE estimates at FMR, we fit a logistic function to the ratio of MCP to AKDE estimates plotted against effective sample size ($\hat{N}$_area_) using the *quantreg* package in R (Koenker 2023). A good fit of the data to a logistic curve would indicate that MCP estimates underestimate AKDE estimates at low $\hat{N}$_area_. Visual examination of Sugarloaf AKDE estimates did not suggest a nonlinear relationship between the 4‐year and full estimates, so we conducted a repeated‐measures ANOVA of the paired estimates from each tortoise using the *rstatix* package in R (Kassambara 2023). A significant test result would indicate that home‐range estimates from the 4‐year subset of data are not equivalent to estimates from the full dataset.

#### 2.3.2 Recursive Burrow Use

We analyzed tortoise burrow use with the *recurse* package in R (Bracis, Bildstein, and Mueller 2018). The function “getRecursionsAtLocations” determined the segments of a tortoise's movement trajectory by connecting the closest dates and added a revisit to each mapped burrow the trajectory intersected. Because each burrow a tortoise used was individually marked, GPS coordinates were consistent across observations within a burrow, and we applied a 1‐m radius to the recursion analysis. We identified the number of unique burrows used by each tortoise; the mean number of visits by each tortoise to each burrow, in which a single visit could include multiple sequential observations within that burrow; the total number of observations each tortoise was recorded in each burrow; and the mean duration each tortoise occupied each burrow during a visit, including those used during hibernation. Absolute duration estimates are imprecise due to the relatively long tracking interval, but we used them to investigate general patterns of use.

#### 2.3.3 Hypotheses and Statistical Analyses

Each hypothesis, statistical test, and final model are listed in Appendix A. We first hypothesized that adult home ranges (throughout, “home range” refers to the full 95% AKDE home ranges) were equivalent between sexes and sites and that ranges were not affected by the number of available burrows (log_e_ transformed). To account for the variation (heterogeneity) among true effects arising from estimated uncertainty in home‐range size within individual tortoises, we used the R package *metafor* (Viechtbauer 2010) to conduct mixed‐effects, within‐study meta‐analyses (Mengersen, Jennions, and Schmid 2013). We used restricted maximum likelihood to estimate the variance of the distribution of true effect sizes, *τ* ^2^, and report how much of the total variability in the effect‐size estimates can be attributed to heterogeneity among the true effects (*I* ^2^; Higgins and Thompson 2002). A complete absence of heterogeneity between individual tortoises would produce *I* ^2^ = 0. We used Knapp‐Hartung adjustments (Knapp and Hartung 2003) to calculate the confidence interval around pooled effects. We used a permutation test to recalculate the *p*‐values of our models based on the test statistics obtained across 1000 permutations of our original dataset to validate the robustness of the final model and to adjust inferences accordingly (Viechtbauer 2010; Harrer et al. 2021). We compared density of the total number of burrows within AKDE home ranges between sites and sexes with generalized least squares regression (GLS) using R package *nlme* (Piniero et al. 2023). We repeated the meta‐analysis for 50% core areas and 50%–95% noncore areas and followed by assessing burrow density versus sex, site, and home‐range portion with repeated‐measures ANOVA. We also compared the relationship of ICU to the number of burrows within core areas and between sites and sexes with a generalized linear model and Gamma distribution with a logarithmic link function (R package *glmmTMB*; Brooks et al. 2017).

We then examined how general burrow use differed between the two sites and sexes. We used GLS to evaluate the hypotheses that the number of burrows used by individual tortoises did not differ between sites, sexes, or relative to the number of burrows available within their 95% home ranges, 50% core areas, and 50%–95% noncore areas. We also used GLS to test the hypothesis that the mean number of visits to individual burrows did not differ between sites or sexes. Next, we tested whether the time tortoises spent within burrows differed by site, sex, or the estimated date that the tortoise entered the burrow. We used package *glmmTMB* to evaluate effects of the predictor variables with a generalized linear mixed‐effects model (GLMM) and Gamma distribution with a log link and with burrow ID and tortoise ID as random effects. We square‐root‐transformed the response variable, the estimated number of days spent within a burrow, to improve normality and dispersion. Looking deeper into how tortoises shared burrows (at any time, not only simultaneously) within overlapping home ranges, we used a generalized linear model with a Poisson distribution in *glmmTMB* to evaluate the hypothesis that burrows were shared equally among sex dyads (female–female, female–male, and male–male) and among sites. The log_10_ of the total number of burrows available within the overlapping portion of each individual dyad's home ranges served as an offset for the count of shared burrows, we included the log_10_ of the total number of observations between individuals of each dyad to control for differences in sample sizes, and we included the log_10_ of the estimated home range overlap to correct for the degree to which sharing burrows was possible.

Next, we tested hypotheses about use of different shelter types relative to (1) how many shelters of each type that tortoises used, (2) the proportion of each type of burrow within their home ranges that tortoises used, (3) the use versus availability of different burrow types in terms of how many times tortoises visited each type of burrow, and (4) the use versus availability of different burrow types in terms of how often tortoises were actually observed in each type of burrow. We assessed whether the number of shelters used (including pallets) varied by shelter type or sex with linear mixed‐effects models in *nlme*, specifying individual tortoise ID as a random effect. For the set of burrows within each tortoise's home range, we determined whether tortoises used a similar proportion of each burrow type and whether that use differed by sex with binomially distributed GLMMs in *lme4* (Bates et al. 2015), again with tortoise ID as a random effect. We assessed whether the number of times tortoises visited different burrow types differed relative to their availability within their home ranges with permutation‐based sign tests (package *phuassess*; Pisani 2016; Fattorini et al. 2017); a tortoise that visited burrows of a certain type only a few times would suggest “avoidance” of that burrow type even if they spent much time inside those burrows. We also used permutation‐based sign tests to assess whether the total number of observations (i.e., tracking relocations) within different burrow types differed relative to their availability; here, multiple relocations of a tortoise in the same burrow (or set of burrows of the same type) indicates “preference” of that burrow type compared to tortoises observed within various burrows of different types during the same tracking occasions. We conducted these analyses separately for FMR and Sugarloaf due to the lack of caliche burrows at Sugarloaf. Lastly, we plotted the number of shelter types used as hibernacula at both sites.

We began each analysis with a global model that included interaction terms among predictors, and we used AICc to compare the global model and various simpler combinations of predictors (Burnham and Anderson 2002). When the final model included nonsignificant interaction terms, we relied on the corresponding additive model for inference (*α* = 0.05; Fieberg and Johnson 2015; Dormann et al. 2018). We evaluated model assumptions (e.g., normality, homoscedasticity) by visual examination of residual plots, *Q*–*Q* plots, and likelihood profile plots in base R, *metafor*, *DHARMa* (Hartig 2024), and *MuMIn* (Bartoń 2014), as applicable. We used DFFITS values, Cooks distances, hat values, and DFBETAS to identify influential cases in meta‐analyses in *metafor* according to Viechtbauer (2010) and Harrer et al. (2021). We conducted pairwise comparisons of marginal means with *emmeans* (Lenth 2023), and we report parameter estimates with 95% confidence intervals in reference to statistical clarity (Dushoff, Kain, and Bolker 2018).

## 3 Results

### 3.1 Comparisons of AKDE With Previous Home‐Range Estimates

**FIGURE 2:**
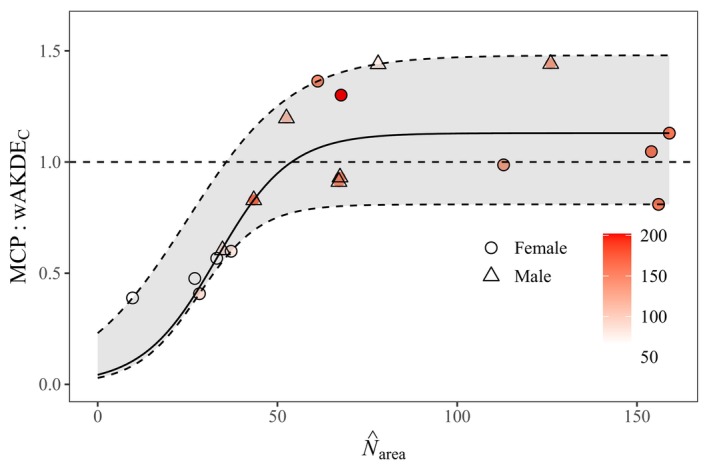
Ratio of 95% minimum convex polygon (MCP) home‐range estimates to 95% optimally weighted autocorrelated kernel density estimates (wAKDE_C_) as a function of effective sample size ($\hat{N}$_area_) for *Gopherus morafkai* at the Florence Military Reservation, Arizona. The solid line represents the fit of a logistic curve to the data with the 95% confidence band in gray. The color gradient represents the absolute sample size (number of relocations).

We tracked 18 tortoises at FMR over 2–4 years and 28–202 observations ($\bar{X}$ = 116.0 ± [SD] 48.84; Table 1; Appendix B). Spatial autocorrelation was common among individual tracking histories: Only six cases met the assumption of IID data, while nine best fit OU and three best fit OUF movement models (Appendix B). Bias caused by spatial autocorrelation caused home ranges calculated with MCP ($\bar{X}$ = 23.9 ± 23.04 ha) to underestimate those calculated by AKDE ($\bar{X}$ = 28.8 ± 34.92 ha) by about 50% at small sample sizes ($\hat{N}$_area_ < 53), which included almost half the tortoises in the study (Figure 2; Appendix B). Only one case (with 79 locations) out of eight with fewer than 127 total locations had an effective sample size ($\hat{N}$_area_ = 78) sufficient not to underestimate the home range.

**TABLE 1:** *Gopherus morafkai* home‐range estimates in Arizona, 2000–2004.

	No. fixes	No. years	Mean sample interval (days)	$\hat{N}$_area_	95% AKDE (ha), 95% CI	50% Core AKDE (ha), 95% CI	PA	ICU	50%–95% AKDE (ha)	Comp
Florence Military Reservation										MCP
^7^Male $\bar{X}$	121.7	3.4	5.4	67.0	32.7	6.4	0.20	2.55	26.4	33.4
Male SD	32.95	0.79	1.12	30.04	17.3–56.4	3.5–10.7	0.040	0.489	26.20	28.95
^11^Female $\bar{X}$	112.5	3.0	6.1	78.3	24.5	5.8	0.23	2.22	20.0	17.9
Female SD	58.03	0.89	2.02	58.98	12.1–43.8	2.8–10.4	0.043	0.421	27.90	17.23
Total $\bar{X}$	116.1	3.2	5.9	73.9	27.7	5.9	0.22	2.35	22.5	23.9
Total SD	48.84	0.86	1.72	48.96	3.6–9.1	3.6–9.1	0.043	0.465	26.65	23.04

	No. fixes	No. years	Mean sample interval (days)	$\hat{N}$_area_	95% AKDE (ha), 95% CI	50% Core AKDE (ha), 95% CI	PA	ICU	50%–95% AKDE (ha)	Comp
Sugarloaf Mountain					AKDE_short_				AKDE_full_	
^5^Male $\bar{X}$	72.0	3.0	8.2	45.1	11.6	2.6	0.22	2.27	9.1	12.6
Male SD	14.16	0.00	1.13	5.21	10.2–13.2	2.2–2.9	0.016	0.167	2.06	3.26
^19^Female $\bar{X}$	98.8	2.9	7.3	54.5	7.5	1.7	0.20	2.68	6.1	7.9
Female SD	54.72	1.33	0.68	34.70	5.0–10.7	0.9–2.7	0.053	0.983	4.19	4.39
Total $\bar{X}$	93.2	2.9	7.5	52.6	8.4	1.8	0.21	2.59	6.7	8.9
Total SD	50.02	1.18	0.85	31.02	6.0–11.4	1.1–2.8	0.048	0.888	4.00	4.55

*Note:* PA, proportion of the 95% home‐range area contained by the 50% core area; Comp = comparison with minimum convex polygon (MCP) estimates from Riedle et al. (2008) at Florence or cumulative AKDE estimates from 1996 through 2005 (AKDE_full_) reported by Averill‐Murray, Fleming, and Riedle (2020) at Sugarloaf. 95% CIs given instead of standard deviations for 95% and 50% AKDE estimates. Superscripts indicate sample sizes.

Abbreviations: AKDE, area‐corrected, optimally weighted, autocorrelated kernel density estimation home range; ICU, intensity of core area use; $\hat{N}$_area_, effective sample size.

We tracked 24 tortoises at Sugarloaf over 1–4 years and 25–158 observations ($\bar{X}$ = 93.2 ± 50.02; Table 1; Appendix C). Home ranges estimated by AKDE from 2000 to 2003 ($\bar{X}$ = 8.6 ± 5.15 ha) slightly underestimated the full 1996–2005 estimates ($\bar{X}$ = 8.9 ± 4.55 ha), but there was no detectable difference in pairwise estimates (AKDE_short_ − AKDE_full_ = −0.2 [−1.3 to 0.9]; Table 1). Model selection between datasets was comparable, with a few exceptions of an OUf movement process selected in the reduced dataset instead of the OU process in the full dataset, and an OU movement process selected over the single case of IID data in the full dataset (Appendix C).

### 3.2 Factors Affecting Home‐Range Size

Home‐range estimates that account for estimation uncertainty differed primarily by population. Home ranges averaged 32.7 ha (95% CI = 17.3–56.4) and 24.5 ha (12.1–43.8) at FMR for males and females, respectively (Table 1). We could not clearly discriminate female and male home‐range sizes due to the large variation in individual estimates (M/F = 1.17 [0.40–2.78]). The volcanic hill occupied 0.7%–33% ($\bar{X}$ = 0.15 ± 0.102) of the seven home ranges that included it. Population‐level home‐range estimates for males and females at Sugarloaf were 11.6 ha (10.2–13.2) and 7.5 ha (5.0–10.7), respectively (Table 1). Here, a difference between males and females was more apparent, but still had a small chance of no real difference (M/F = 1.45 [0.98–2.27]). Between sites, tortoises at FMR had larger home ranges (FMR/Sugarloaf = 3.07 [1.59–5.24]), which held for all comparisons between sexes (FMR_male_/Sugarloaf_male_ = 2.81 [1.35–4.83]; FMR_male_/Sugarloaf_female_ = 4.19 [1.81–8.00]; FMR_female_/Sugarloaf_female_ = 3.16 [1.26–6.23]) except for slight uncertainty between FMR females and Sugarloaf males (2.12 [0.94–3.79]).

**FIGURE 3:**
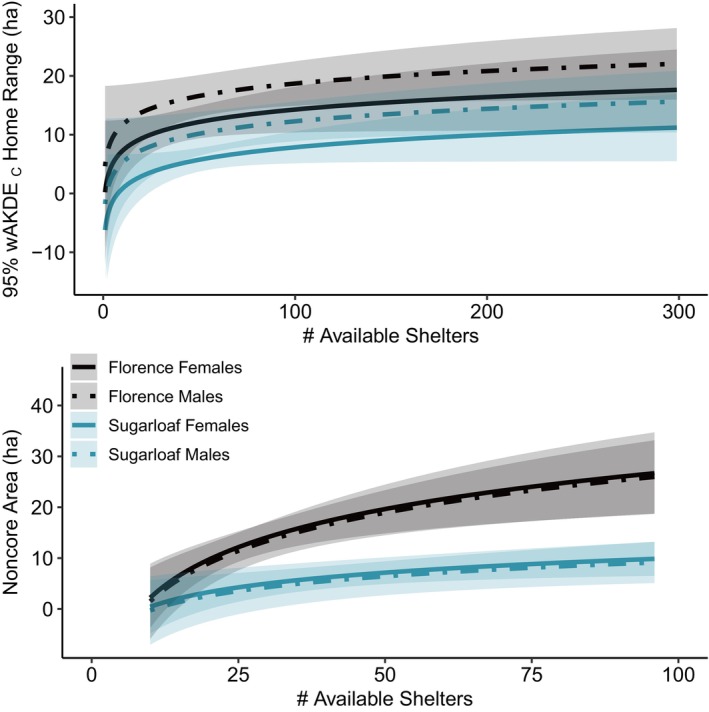
Relationships between 95% optimally weighted autocorrelated kernel density estimates (wAKDE_C_; top) and 50%–95% noncore home‐range areas (bottom) versus the number of available shelters within *Gopherus morafkai* populations at the Florence Military Reservation and Sugarloaf Mountain, Arizona, 2000–2003. Results for 50% core areas were qualitatively similar to 95% wAKDE_C_, with smaller effects for the slope and differences between site and sex (see Appendix A.3).

Initial meta‐analysis suggested that sex and an interaction between population and the number of available burrows affected home‐range size (ΔAICc > 7.27). Removal of three influential cases (FMR M#403, M#419, and F#410) resulted in a selected model with an interaction between sex and the number of available burrows (ΔAICc > 0.21), but the interaction was nonsignificant so we relied on the additive model for inference. Female tortoises had smaller home ranges than males (F–M = −4.4 ha [−8.2 to −0.7]), and tortoises at FMR used larger home ranges than those at Sugarloaf (FMR–Sugarloaf = 6.4 ha [3.2–9.7]). The permutation test also indicated that home‐range size increased modestly with the number of burrows available (${\hat{\beta }}_{\ln\left(\#\text{burrows}\right)}$ = 3.06 [−0.14 to 6.25]; Figure 3; Appendix A.1). Between‐tortoise heterogeneity variance was *τ* ^2^ = 10.8 (10.0–57.0), with *I* ^2^ = 95.8% (95.4%–99.2%) highlighting the substantial heterogeneity among tortoises. The influential cases in the original model essentially caused home‐range size to increase more rapidly with the number of burrows at FMR than at Sugarloaf.

**FIGURE 4:**
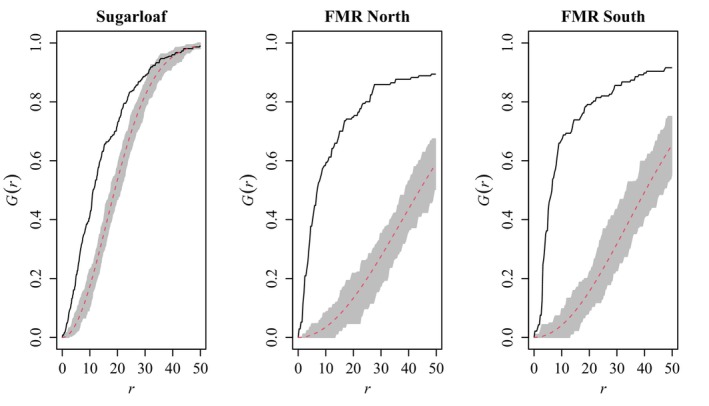
Nearest‐neighbor distance distribution functions for burrows at Sugarloaf and the north and south subpopulations at the Florence Military Reservation (FMR). Values are probabilities of nearest‐neighbor burrow distances as a function of distance *r* (in m). Empirical black lines above the dashed theoretical curves and associated 5% simulation envelopes indicate that burrow nearest‐neighbor distances are shorter within each population than expected under complete randomness, consistent with clustering.

The abundance and distribution of burrows differed markedly between populations. Home ranges at FMR contained an average of 63.4 (± 34.03) burrows, while Sugarloaf home ranges contained 82.0 (± 54.36) burrows (Table 2; Appendices D, E). Burrow distribution was clustered within both the FMR north and south subpopulations and at Sugarloaf (*R* = 0.39 ± 0.040, 0.30 ± 0.038, and 0.70 ± 0.024, respectively; all *p* < 0.001). The median distance between nearest burrows (i.e., at *G*_*r*_ = 0.5) was 6.7 m, 6.4 m, and 11.1 m at FMR north/south and Sugarloaf, respectively (Figure 4). Pairwise comparisons revealed no clear difference in home‐range burrow density between sexes, but FMR home ranges contained ~33% (0.23–0.47) the densities of burrows as Sugarloaf home ranges (Appendix A.2).

**TABLE 2:** Numbers of shelters available within *Gopherus morafkai* home ranges, core areas, and noncore areas in Arizona, 2000–2004.

	95% wAKDE_C_					50% Core areas						50%–95% Noncore areas					
	Total	Caliche	Rock	Soil	Rat	Total	Density (ha^−1^)	Caliche	Rock	Soil	Rat	Total	Density (ha^−1^)	Caliche	Rock	Soil	Rat
*Florence Military Reservation*																	
^7^Male $\bar{X}$	68.6	45.1	8.0	6.0	9.4	25.0	5.4	15.7	2.9	2.6	3.9	43.6	2.3	29.4	5.1	3.4	5.6
SD	30.53	20.24	13.01	5.54	7.93	12.86	3.32	9.55	5.98	4.08	5.27	26.14	1.49	20.18	8.63	3.21	5.97
^11^Female $\bar{X}$	60.2	33.1	9.8	6.6	10.6	25.6	8.0	14.7	4.3	2.7	3.9	34.5	2.5	18.4	5.5	3.9	6.7
SD	37.13	23.87	11.83	5.94	8.00	8.76	4.53	11.27	6.50	3.00	5.19	31.30	1.24	16.25	8.50	4.25	6.96
Total $\bar{X}$	63.4	37.8	9.1	6.4	10.2	24.9	6.8	14.4	3.9	2.8	3.8	38.6	2.4	23.2	5.7	3.8	6.0
SD	34.03	22.72	11.96	5.63	7.76	10.25	4.22	10.14	6.28	3.38	5.19	29.73	1.34	18.59	8.44	3.90	6.52
*Sugarloaf Mountain*																	
^5^Male $\bar{X}$	99.4		85.8	13.2	0.4	27.8	12.3		24.6	3.0	0.2	71.6	7.8		61.2	10.2	0.2
SD	25.39		25.95	2.17	0.89	18.32	9.65		17.64	2.65	0.45	30.76	2.43		28.67	2.49	0.45
^19^Female $\bar{X}$	77.4		67.8	9.2	0.4	23.9	16.6		20.7	3.2	0.0	53.4	10.3		47.1	5.9	0.4
SD	59.38		56.67	5.47	0.60	21.38	9.51		19.30	3.68	0.00	39.71	6.07		38.78	3.61	0.60
Total $\bar{X}$	82.0		71.6	10.0	0.4	24.8	15.7		21.5	3.2	0.0	57.2	9.8		50.0	6.8	0.3
SD	54.36		51.83	5.20	0.65	20.46	9.50		18.66	3.43	0.20	38.15	5.56		36.80	3.80	0.56

*Note:* wAKDE_C_ = area‐corrected, optimally weighted, autocorrelated kernel density estimation home range; Rat = packrat. Superscripts indicate sample sizes.

As for home ranges, tortoise core areas at FMR were larger than those at Sugarloaf (FMR–Sugarloaf = 1.8 ha [0.5–3.0]; Table 1; Appendices A.3, B, C). The permutation test also indicated that core area size increased with the number of burrows available, although less strongly than 95% home ranges (${\hat{\beta }}_{\text{sqrt}\left(\#\text{burrows}\right)}$ = 0.37 [−0.03 to 0.77]), and was greater for males than females (F–M = −1.2 ha [−2.5 to 0.1]; Appendix A.3). Between‐tortoise heterogeneity variance was *τ* ^2^ = 1.7 (2.4–12.4), with *I* ^2^ = 99.3% (99.5%–99.9%) again indicating substantial heterogeneity among tortoises. The size of core areas averaged just over 20% of the total home ranges at both FMR (${\bar{X}}_{\mathrm{PA}}$ = 0.22 ± 0.043) and Sugarloaf (${\bar{X}}_{\mathrm{PA}}$ = 0.21 ± 0.048; Table 1; Appendices B, C), but PA was uncorrelated with home‐range size at both sites (*r*_*FMR*_ = −0.05 [−0.50 to 0.43]; *r*_*Sugarloaf*_ = 0.34 [−0.08 to 0.65]).

Model selection in the initial meta‐analysis of noncore areas included sex and an interaction term between population and the number of available burrows (ΔAICc ≥ 14.68). Removal of three influential cases (FMR M#403, M#413, and F#501) resulted in selection of the same model (ΔAICc > 2.21). Under the latter analysis, tortoise noncore areas averaged 22.5 ± 26.65 ha at FMR and 6.7 ± 4.00 ha at Sugarloaf (Table 1; Appendices B, C). Noncore areas were larger at FMR than at Sugarloaf, and they increased more rapidly with the number of burrows at FMR than at Sugarloaf; there was not a clear difference between sexes (Figure 3; Appendix A.4). Between‐tortoise heterogeneity variance was *τ* ^2^ = 23.9 (15.6–41.3), with *I* ^2^ = 99.6% (99.4%–99.8%).

**FIGURE 5:**
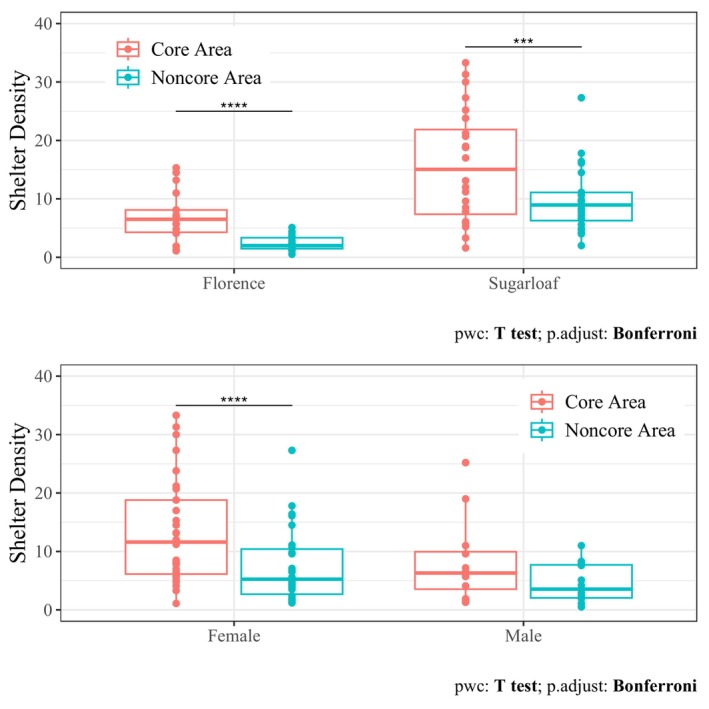
Shelter density within 50% core areas and 50%–95% noncore areas for *Gopherus morafkai* at the Florence Military Reservation and Sugarloaf (top) and between females and males across sites (bottom), 2000–2003. Asterisks indicate significantly different pairwise comparisons.

Burrow densities varied within home ranges and affected the intensity at which tortoises used their core areas. Burrow densities at FMR averaged 6.8 burrows/ha (±4.22) within core areas and 2.4 burrows/ha (±1.34) within noncore areas (Table 2; Appendix D). Corresponding burrow densities at Sugarloaf averaged 15.7 burrows/ha (±9.50) and 9.8 burrows/ha (±5.56), respectively (Table 2; Appendix E). Greater burrow densities in core areas than noncore areas at both FMR and Sugarloaf (Figure 5) was driven by differences between areas for females but not for males (Figure 5; Appendix A.5). Intensity of core area use (ICU) averaged 2.35 ± 0.465 and 2.59 ± 0.888 at FMR and Sugarloaf, respectively (Table 1; Appendices B, C). ICU decreased with the number of shelters within the core area (${\hat{\beta }}_{\#\text{burrows}}$ = −0.006; −0.010 to −0.002), but it did not clearly differ by site or sex (Appendix A.6). Across the range of core area burrows available, predicted ICU decreased from 2.89 with 1 burrow to 1.54 with 98 burrows.

### 3.3 Factors Affecting Burrow Use

Tortoises at FMR used an average of 29.4 (±12.89) shelters within their overall home ranges, 17.6 (±8.07) within their core areas, and 11.7 (±6.37) within noncore areas (Appendix F); overall, tortoises used 200 burrows (excluding pallets) during the study. Corresponding numbers of shelters used by tortoises at Sugarloaf averaged 26.8 (± 11.92), 14.9 (± 8.67), and 11.8 (± 5.90) for overall home ranges, core areas, and noncore areas, respectively (Appendix G), and they used a total of 265 burrows. The number of burrows used by tortoises was not clearly related to the number available within home ranges or noncore areas; likewise, the number of burrows used did not clearly differ by site or sex within overall home ranges, core areas, or noncore areas (Appendix A.7–A.9). However, within core areas, the number of burrows tortoises used increased with the number available (${\hat{\beta }}_{\#\text{available burrows}}$ = 0.13 [0.05–0.21]; Appendix A.8). Females tended to repeatedly visit individual burrows more often than males at Sugarloaf, but sexes did not clearly differ at FMR (Appendix A.10).

**FIGURE 6:**
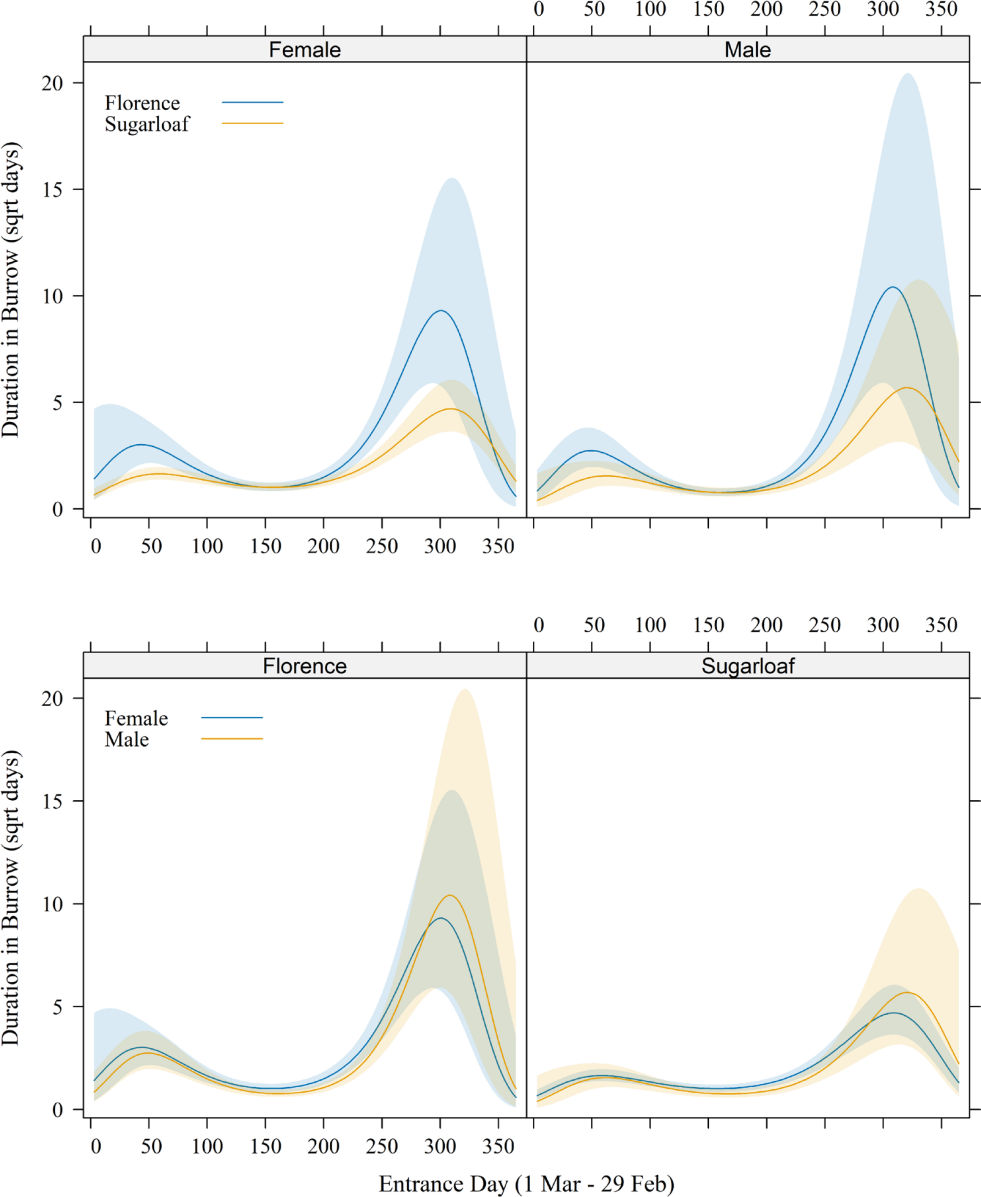
Duration of visits within individual burrows by *Gopherus morafkai* relative to sex and the date that tortoises entered burrows at Florence Military Reservation and Sugarloaf Mountain, 2000–2003. Both panels depict results from the same model, with the top panel emphasizing differences in sites for each sex and the bottom panel emphasizing differences in sex at each site. Day 1 = 1 March.

Time spent within burrows generally corresponded with the activity season. Male and female tortoises spent 15.4 ± 40.05 days and 13.7 ± 31.76 days within each burrow at FMR, respectively, and 14.6 ± 43.07 days and 12.1 ± 25.88 days within each burrow at Sugarloaf. After accounting for random effects of tortoise ID and shelter ID, time spent in burrows decreased for tortoises entering burrows as the activity season progressed from spring through summer before increasing as tortoises entered winter hibernation, but tortoises at FMR spent even more time in their hibernacula than at Sugarloaf (Figure 6; Appendix A.11). Otherwise, female tortoises spent about 22% more time on average inside burrows than males (F:M = 1.22 [1.01–1.47]), although the pairwise‐comparison confidence interval and the effects graph indicated that this may have been a difference of minimal biological significance (Figure 6).

Overall, pairs of tortoises overlapped AKDE home ranges similarly at FMR ($\bar{X}$ = 0.39 ± 0.212 SD) and Sugarloaf ($\bar{X}$ = 0.34 ± 0.213). Available burrows within the overlapping portion of home ranges tended to be fewer at FMR ($\bar{X}$ = 28.5 ± 18.62) than at Sugarloaf ($\bar{X}$ = 31.5 ± 26.32), while tortoises at FMR tended to share more burrows ($\bar{X}$ = 2.0 ± 2.17) than at Sugarloaf ($\bar{X}$ = 0.9 ± 1.43). Pairs of tortoises at FMR shared 1.72 times (1.24–2.37) as many burrows on average than pairs at Sugarloaf, but the rate of sharing increased with the number of combined fixes for each pair more rapidly at Sugarloaf than at FMR (Appendix A.12). Female:female pairs shared 23% fewer burrows (0.46–0.97) than female:male pairs, while other dyad comparisons did not clearly differ (Appendix A.12). Although we do not present the results, partitioned by year and using unique pairs of individual tortoises as a random effect, relationships between shared burrows and the continuous variables and with site were qualitatively similar to the results above, but the number of shared burrows used/pair were too low and variable for clear differences between dyad types to emerge. The number of shared burrows in 2000 tended to be greater than in 2002 and 2003, but all estimates were highly variable.

### 3.4 Shelter Use Relative to Shelter Type

Tortoises used more pallets (16.1 ± 11.07) than all other shelter types at FMR, they used more caliche burrows on average (6.3 ± 4.38) than rock (2.3 ± 3.48) or soil burrows (2.8 ± 2.71), and they used an intermediate number of packrat burrows (3.4 ± 3.93), but use of different shelter types did not clearly differ between sexes (${\hat{\beta }}_{\mathrm{sex}}$ = 0.11 [−1.65 to 1.88]; Table 3; Appendix A.13). Among permanent burrow types (i.e., excluding pallets), tortoises used relatively fewer of the available caliche burrows (17%) than all other types at FMR and a lower proportion of the available rock burrows (30%) than soil burrows (50%), again with no clear difference between sexes (${\hat{\beta }}_{\mathrm{sex}}$ = 0.10 [−0.58 to 0.78]; Appendix A.14).

**TABLE 3:** Numbers of shelters used by type by *Gopherus morafkai* in Arizona, 2000–2004.

	95% wAKDE_C_						50% Core areas						50%–95% Noncore areas					
	Caliche	Rock	Soil	Rat	Total	Pallet	Caliche	Rock	Soil	Rat	Total	Pallet	Caliche	Rock	Soil	Rat	Total	Pallet
*Florence Military Reservation*																		
^7^Male $\bar{X}$	8.6	2.0	2.1	3.7	16.4	15.7	4.7	1.1	1.0	2.4	9.3	7.0	3.9	0.9	1.1	1.3	7.1	8.7
SD	5.38	3.46	2.54	3.64	6.21	11.04	3.64	2.61	1.83	2.37	5.99	3.74	2.61	1.86	1.46	1.98	1.95	9.53
^11^Female $\bar{X}$	4.9	2.5	3.2	3.2	13.8	16.4	3.5	2.1	2.3	2.5	10.4	8.4	1.4	0.5	0.9	0.7	3.5	8.0
SD	3.08	3.64	2.86	4.26	5.10	11.62	3.30	3.21	2.80	3.47	3.20	6.22	1.57	0.82	0.83	1.19	2.62	6.26
Total $\bar{X}$	6.3	2.3	2.8	3.4	14.4	16.1	3.7	1.8	1.9	2.4	9.8	7.9	2.3	0.6	1.0	0.9	4.6	7.1
SD	4.38	3.48	2.71	3.93	5.40	11.07	3.24	3.00	2.52	3.01	4.42	5.46	2.39	1.32	1.12	1.51	2.87	5.51
*Sugarloaf Mountain*																		
^5^Male $\bar{X}$		11.4	1.6	0.2	13.2	13.8		6.6	0.4	0.2	7.2	8.2		4.8	1.2	0.0	6.0	5.6
SD		3.05	1.14	0.45	2.05	10.06		2.70	0.55	0.45	2.59	8.58		1.92	1.10	0.00	1.58	1.52
^19^Female $\bar{X}$		11.6	2.3	0.1	13.9	12.7		6.5	1.4	0.0	7.9	6.8		5.1	0.8	0.1	6.0	5.9
SD		6.54	1.91	0.32	6.78	9.05		4.44	1.71	0.00	4.77	6.78		3.64	1.01	0.32	3.83	3.67
Total $\bar{X}$		11.5	2.1	0.1	13.8	13.0		6.5	1.2	0.0	7.8	7.1		5.0	0.9	0.1	6.0	5.8
SD		5.93	1.78	0.34	6.06	9.05		4.09	1.59	0.20	4.36	7.01		3.32	1.02	0.28	3.45	3.31

*Note:* wAKDE_C_ = area‐corrected, optimally weighted, autocorrelated kernel density estimation home range; Rat = packrat. Totals do not include pallets. Superscripts indicate sample sizes.

**FIGURE 7:**
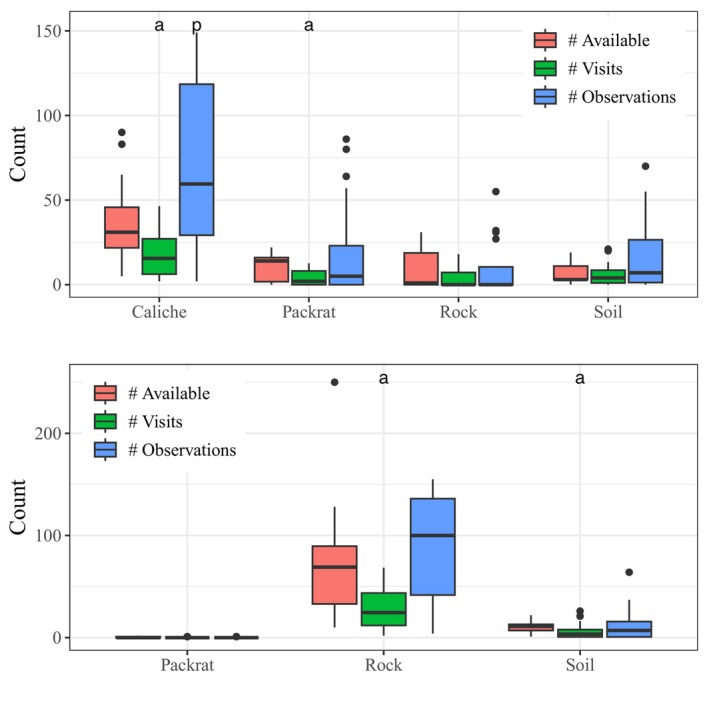
Number of burrows of different types available within *Gopherus morafkai* home ranges, the number of visits to each burrow, and the number of observations within each burrow at the Florence Military Reservation (top) and Sugarloaf Mountain (bottom). Visits or observations within burrow types for which permutation‐based sign tests indicated avoidance (a) or preference (p) are indicated above the corresponding bars.

**FIGURE 8:**
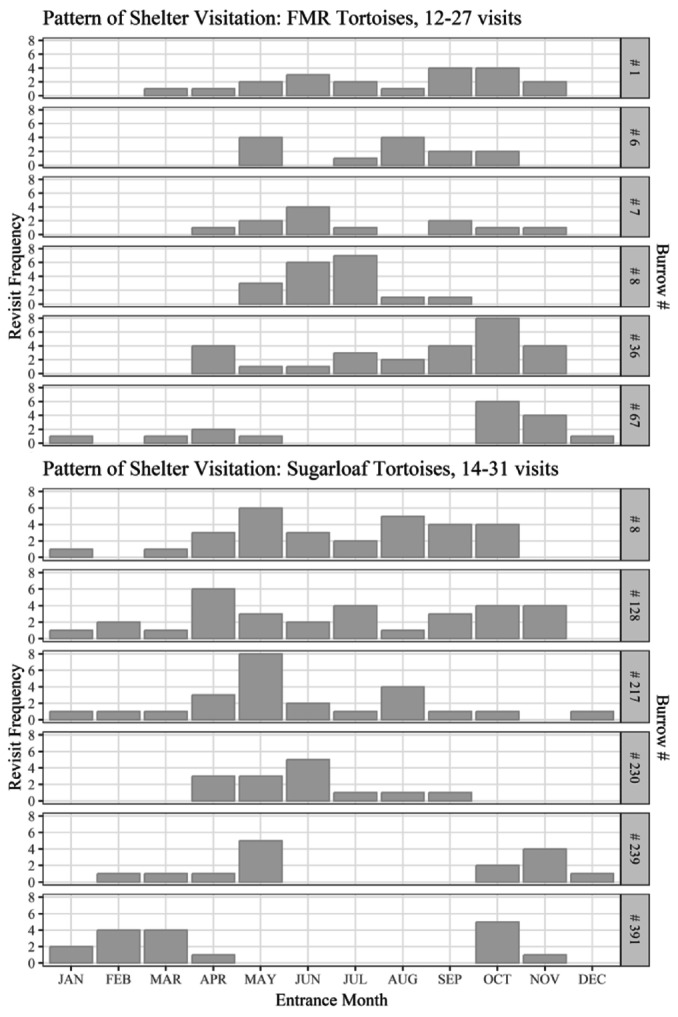
Temporal pattern of visitation for the most‐frequently visited burrows by *Gopherus morafkai* at the Florence Military Reservation (top; all caliche except rock burrow #67) and at Sugarloaf Mountain (bottom; all rock). From top to bottom, burrows were used by 5, 3, 3, 4, 5, and 2 tortoises, respectively, at Florence, and by 4, 2, 3, 1, 3, and 2 tortoises at Sugarloaf.

Fewer than 21% of tortoises visited caliche and packrat burrows in proportion to the availability of those burrow types, while they generally visited soil and rock burrows in proportion to their availability (Figure 7; Appendix A.15). However, at the observation level, tortoises preferred caliche burrows, with 83% of tortoises found inside caliche burrows at a greater proportion than their availability, while we observed tortoises inside soil, rock, and packrat burrows in proportion to their availability (Figure 7; Appendix A.16). The six most‐visited burrows (5 caliche, 1 rock) were visited 12–27 times by 2–5 tortoises. Seasonal patterns of use suggested that certain burrows were preferred at some times of the year over others (Figure 8). For example, two tortoises used rock burrow #67, but never during the middle of summer: F#220 and M#419 hibernated in this burrow in 2001 and 2003, respectively. In contrast, caliche burrows #6 and #8 were used exclusively in summer by three and four tortoises, respectively. The remaining three burrows were used throughout the year: #1 by five tortoises, including 3 years of hibernation by M#406; #7 by three tortoises, including 1 year of hibernation by F#210; and #36 by five tortoises, including 4 years of hibernation by F#404.

At Sugarloaf where caliche was not present, tortoises used many more rock burrows (11.5 ± 5.93) and pallets (13.0 ± 9.05) than packrat (0.1 ± 0.34) and soil burrows (2.1 ± 1.78), and they used more soil burrows than packrat burrows (Table 3; Appendix A.17). They used 16%–26% of the burrows in each type within their home ranges, and differences in burrow use did not clearly differ between sexes either in terms of absolute or relative use (${\hat{\beta }}_{\text{number}/\mathrm{sex}}$ = 0.07 [−0.28 to 0.42]; ${\hat{\beta }}_{\%\text{type}/\mathrm{sex}}$ = −0.57 [−1.49 to 0.35]; Appendices A.17, A.18). Tortoises visited fewer soil and rock burrows than expected, with only ~25% visiting these burrow types in proportion to their availability within their home ranges (Figure 7; Appendix A.19). However, we observed tortoises within all burrow types in proportion to their availability (Figure 7; Appendix A.20). The six most‐visited burrows (all rock) were visited 14–31 times by 1–4 tortoises. As at FMR, tortoises preferred certain burrows at some times of the year over others (Figure 8). Only F#66 used burrow #230, exclusively during the warmer months of the year; she produced eggs in 3 years of the study and used this burrow shortly preceding oviposition each of these years, suggesting a possible preferred nesting site. Burrows #239 and #391 were used by three and two tortoises, respectively, but never in the summer; F#58 hibernated in burrow #239 all 4 years, and F#72 hibernated in burrow #391 for two winters. Finally, burrows #8, #128, and #217 were used throughout the year: #8 by four tortoises, including 1 year of hibernation by F#57; #128 by two tortoises, including 3 years of hibernation by F#68; and #217 by three tortoises, including hibernation by F#17 for one winter.

**FIGURE 9:**
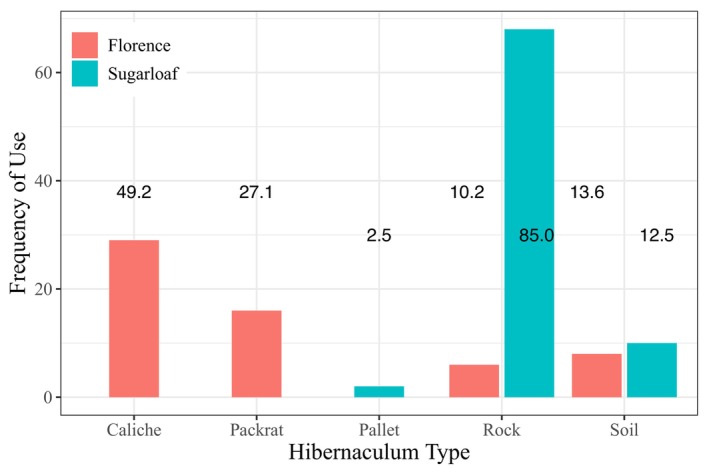
Frequency that each shelter type was used as a hibernaculum by *Gopherus morafkai* at the Florence Military Reservation and Sugarloaf Mountain. Relative frequencies within sites are indicated for each hibernaculum type.

Tortoises had clear preferences for specific burrow types as hibernacula. Tortoises at FMR primarily used caliche (49.2%) or packrat burrows (27.1%) as hibernacula, while Sugarloaf tortoises predominately used rock burrows (85.0%; Figure 9). Except for the two pallets each used by a different tortoise at Sugarloaf, tortoises reused hibernacula during multiple winters up to the maximum possible four winters studied at each site ($\bar{X}$ = 1.5 winters/tortoise ±0.84). Different tortoises used the same burrow for hibernation twice during the study. At FMR, two females hibernated in the same caliche burrow in two winters, sharing it during one of those. Two Sugarloaf females hibernated in the same rock burrow in 2003 that one of them also used during the two prior winters. Twice, tortoises at Sugarloaf spent at least part of their hibernation in a pallet with minimal insulation. Female #69 spent roughly 5 months in a pallet below a shrub from November 2000 through March 2001. Female #86 spent 2–3 weeks in a pallet below a shrub in November 2001 before moving to a soil burrow through February 2002.

## 4 Discussion

### 4.1 Application of AKDE

A common justification for the use of MCP or KDE that many publications on animal home range continue to cite is that they facilitate comparisons with previous estimates in the literature (e.g., Gamblin et al. 2024; Garrido‐Priego et al. 2024; Nguyen, Halstead, and Todd 2024; Pyott et al. 2024). However, the selection of OU or OUF/f models, which correct for autocorrelation, as the best fit for most tortoises in this study demonstrates the inadequacy of conventional estimation: Data from only six of 44 tortoises across both study sites met the IID assumption inherent to MCPs and KDEs. Had we simply estimated home ranges with MCP at Sugarloaf to compare with previous estimates from FMR, accurate results and conclusions may have been obscured due to inconsistencies in bias produced by MCP (Figure 2). Applying even an arbitrary minimum sample‐size threshold for “reliable” MCP estimates would have been unreliable, given the variation in absolute sample size with $\hat{N}$_area_, including, for example, an underestimated MCP for the tortoise with the second highest number of observations at FMR (*n* = 163 vs. $\hat{N}$_area_ = 43.4; Figure 2).

These results highlight the importance of interpreting conclusions from home‐range studies using older methods with caution. This is especially true for home‐range overlap, as individual biases from autocorrelated data are directly propagated and magnified into overlap estimates (Winner et al. 2018). Failure to meet the IID assumption also would have led to underestimated KDEs at low $\hat{N}$_area_ because KDE is only statistically optimal when the tracking schedule is so coarse that the data appear uncorrelated in time or when animals are tracked for much longer than the timescale over which any autocorrelation persists (Fleming et al. 2015; Noonan et al. 2019). Unfortunately, Ribeiro et al. (2024) did not find the use of more appropriate and updated methods in any study of freshwater turtle home ranges through 2022 even though conventional methods have been shown to underestimate home‐range areas across dozens of species from various taxonomic groups (Noonan et al. 2019, 2020; Averill‐Murray, Fleming, and Riedle 2020).

At the same time, the lack of systematic differences in pairwise estimates at Sugarloaf indicated that our AKDE estimates over 4 years were unbiased, although they were more variable, relative to estimates from longer durations and larger sample sizes for the same tortoises. Similarly, cumulative and marginal mean annual AKDE home ranges over up to 6 years were comparable in size for Gila Monsters *Heloderma suspectum* (Stalker et al. 2023). AKDE estimates from relatively short sampling durations (e.g., subsets of longer datasets) still reliably predict future space use, with wider confidence intervals at lower *N̂*_area_ tending to compensate for any positive bias (Noonan et al. 2019; Averill‐Murray, Fleming, and Riedle 2020).

Finally, given the net underestimation of MCP estimates compared to AKDE estimates at FMR, simply comparing recent AKDE estimates from Sugarloaf with previous MCP estimates from FMR would have reduced the actual difference in estimated home ranges between the two sites. We found that population‐level AKDE home ranges at FMR were 1.6–5.2 times larger than those at Sugarloaf after correcting for autocorrelation and accounting for uncertainties in the individual estimates according to Fleming et al. (2022). These estimates compensated for different movement behaviors (i.e., uncorrelated locations within the home range [IID] vs. correlated movements with a tendency to remain in a particular area [OU] vs. correlated movements within a particular area plus periods of time when the animal tends to show directional persistence [OUF]; Calabrese, Fleming, and Gurarie 2016) and the slightly different sampling schedules between populations while retaining statistical precision and power (Fleming et al. 2022), and they provide a solid basis of comparison for future studies. We suspect that broad inferences may be made from gross patterns of simple relationships of uncorrected home‐range estimates in many cases, especially when sample sizes are large. However, drawing inferences from specific parameters derived from MCP‐ or KDE‐based estimates, such as comparing the slopes of home range–body mass relationships across different taxa, can be problematic (e.g., Perry and Garland Jr. 2002; Tamburello, Côté, and Dulvy 2015; Slavenko et al. 2016; Todd and Nowakowski 2021). For example, home range–body mass relationships derived from conventional KDE estimates did not differ from linear scaling for either mammalian herbivores or carnivores, but correction for autocorrelation with AKDE resulted in a scaling exponent significantly > 1; the scaling of the relationship changed substantially at the upper end of the mass spectrum and would have affected naïve inferences (Noonan et al. 2020). Similar effects may be true for past taxonomic comparisons of home range–body mass relationships.

### 4.2 Factors Affecting Home‐Range Size

Use and availability of burrows drive space use of *G. morafkai*. Despite substantial individual variation, all measures of home‐range size (95% home ranges, 50% core areas, 50%–95% noncore areas) increased slightly with the number of burrows available to tortoises, and all were greater at FMR than at Sugarloaf. Tortoises at both sites used the same number of burrows on average in each portion of their home ranges, so larger home ranges at FMR appeared to compensate for having only about one third the density of burrows as home ranges at Sugarloaf. In addition, core‐area size was uncorrelated with home‐range size at both FMR and Sugarloaf, indicating that the size of tortoises' core areas varied depending on the distribution of resources (i.e., burrows) within their home ranges (Seaman and Powell 1990).

Helodermatid lizards exhibited similar patterns in home‐range size. Greater resource availability allowed individual *H. suspectum* in a subsidized environment to have up to 66% smaller home ranges than individuals at natural sites (Stalker et al. 2023). In contrast, degraded, fragmented habitat with fewer resources may explain why Guatemalan Beaded Lizards *Heloderma charlesbogerti* have larger home ranges than other helodermatids (Ariano‐Sánchez et al. 2020).

Models have shown that the most important factor determining quality, efficiency, resource content, and spatial distribution of home ranges was the extent to which resources were clumped on a landscape (Mitchell and Powell 2004). Accordingly, burrows, as an important resource for *G. morafkai* , were clustered at both FMR and Sugarloaf, but about twice as much so at FMR, especially due to the association of caliche caves with incised arroyos. Burrow density was greater within core areas than noncore areas at both populations, and tortoises with access to fewer burrows in their core areas used those areas more intensively than those with more available burrows.

*Gopherus agassizii* burrows were also clustered at two populations in California, although they more closely approximated a random distribution than at FMR and Sugarloaf (*R* = 0.85–0.90 [Duda, Kyzysik, and Meloche 2002] compared to 0.30–0.70). Spatiotemporal predictability of burrows strongly reinforces site fidelity of individual desert tortoises (Duda, Kyzysik, and Meloche 2002), as has been observed for annual home ranges at multiple *G. morafkai* populations (Sullivan et al. 2016; Averill‐Murray, Fleming, and Riedle 2020). Likewise, *H. suspectum* , which is broadly sympatric with *G. morafkai* , also exhibits high fidelity to burrows and maintains relatively stable home ranges across years (Stalker et al. 2023). Strong site fidelity in areas with known shelters is advantageous in reducing thermal risk and in economizing energy expenditure in a harsh climate, because it is energetically more efficient to reside in an area that produces reliable forage under favorable growing conditions—and where good shelter sites are available under all conditions—and remain there than it is to roam the desert in a nomadic fashion (Duda, Kyzysik, and Meloche 2002). *Heloderma charlesbogerti* go further in actually contracting their home ranges during the seasonal dry season when resources are extremely scarce, evidently as a survival strategy used to endure arid environmental conditions (Ariano‐Sánchez et al. 2020).

Leveraging the data from both populations and accounting for availability of burrows, we found that females had smaller home ranges and core areas than males, which contrasts with the inconclusive findings from average home‐range estimates alone. This result occurred despite the similar total burrow densities within male and female home ranges. However, females tended to concentrate their core activity in areas with higher burrow density, while burrow density was relatively even between male core and noncore areas. Maintaining fidelity to smaller core areas than males may be especially important for females for whom reproductive success of previous years is not predictive of the quality of resources within a patch in upcoming years, in which case resettlement or broader space use is unlikely to yield benefits that outweigh its risks (Stalker et al. 2023). Rather than exploring additional space to find new areas for better forage, *G. morafkai* females at Sugarloaf skipped reproduction in years they could not obtain the necessary body condition (Averill‐Murray, Christopher, and Henen 2018).

An alternative, not mutually exclusive, explanation for males having larger home ranges than females is that males range across larger areas in search of females with whom to mate (e.g., Boglioli, Guyer, and Michener 2003; Heinsohn et al. 2024). For example, home ranges (100% MCP) and core areas (50% MCP) of male *G. agassizii* were on average 65% and 73% larger than those of females, respectively. Female home ranges and core areas overlapped with more males than did males with males, and the extent of overlap was greater between males and females than between females (Harless et al. 2009). Burrow use influenced the size of core areas for both sexes, but it did not affect the overall home‐range size (Harless et al. 2009). In contrast, even though the number of overlapping individuals was not quantified due to the limit of potential neighbors of tortoises on the periphery of the Sugarloaf study site, the magnitude of home‐range overlap between males and females did not differ from overlap between males (Averill‐Murray, Fleming, and Riedle 2020).

### 4.3 Burrow Use and Sharing

Burrow use differed between sexes and populations in various ways. For example, female tortoises revisited individual burrows more often than males at Sugarloaf, but not at FMR, and females generally appeared to spend at least a little more time overall in burrows than males at both sites. These differences in burrow visitation may relate to nesting as females revisit preferred nest sites, with less apparent differences between sexes at FMR because males have fewer burrows to choose from, thereby equalizing revisit rates.

Time spent within burrows also may relate to reproduction. Even though responses were highly variable, females tended to spend more time in burrows in the summer (when they would be nesting) and less time during winter than males (Figure 6). Female *G. morafkai* were more active and foraged more than males during winter at Sugarloaf, FMR, and two other populations (Bailey, Schwalbe, and Lowe 1995; Sullivan et al. 2014), presumably given strong reproductive motives. Hibernation duration translates directly to reproductive output, with early emerging females more likely to reproduce than later‐emerging females, although why females would choose to emerge later in the spring is perplexing given their ability to develop ovarian follicles quickly from winter and spring forage (Averill‐Murray, Christopher, and Henen 2018).

It is not immediately apparent why tortoises would spend more time in hibernation at FMR than at Sugarloaf (Figure 6), but it could relate to differences in spring forage availability between sites. We did not measure spring plant production at our sites, but the Lower Colorado River Valley Subdivision of the Sonoran Desert (e.g., most of FMR) typically has lower winter rainfall than the Arizona Upland Subdivision (e.g., Sugarloaf; Turner and Brown 1982). Primary production, and thus food availability for tortoises, is directly related to precipitation (Webb et al. 1978; Oftedal 2002), so tortoises of both sexes at FMR may be less conditioned to emerge from hibernation as early as tortoises at Sugarloaf if foraging opportunities are less reliable. Even though females were more active and foraged more than males at both Sugarloaf and FMR, Sullivan et al. (2014) reported greater overall winter activity at Sugarloaf (5.1%) than at FMR (1.5%), a difference we confirmed with a heterogeneity *Χ* ^2^ test (Zar 1996) for the 4 years of concurrent study (*Χ* ^2^ = 13.162; *p* = 0.004).

While our focus was on the importance of shelter sites, other important resources also affect tortoise movements and space use. Tortoises at FMR subsequently were found to select habitat with greater canopy cover, absence of cattle activity, and closer proximity to washes and low‐traffic gravel roads than was available across their home ranges (Grandmaison, Ingraldi, and Peck 2010). Three of these factors relate to burrow use. We have already discussed the relationship of caliche caves to washes, but canopy cover provides additional thermal protection for upland burrows or tortoises seeking shade on the surface (Burge 1978), and cattle can damage or destroy burrows and shrubs used for shelter by tortoises (Berry 1978; Avery and Neibergs 1997). Tortoises also appear to select burrows relative to their degree of nutritional needs as seen by *G. agassizii* in habitat with less abundant forage using burrows closer to nutritious forage plants than expected at random and by tortoises in habitat with abundant forage using burrows more sheltered by large perennials than expected (Nafus et al. 2022).

Finally, even though the number of burrows shared between tortoises increased as the overlap between their home ranges increased, patterns of burrow sharing differed by population and sex. Given the lower number of burrows available at FMR, pairs of tortoises at FMR shared 72% more burrows on average than pairs at Sugarloaf. In addition, pairs of females shared 33% fewer burrows than female–male pairs across both sites. Previously, we found that gravid females at Sugarloaf were less likely to overlap annual home ranges with other gravid females than were pairs of nongravid females (Averill‐Murray, Fleming, and Riedle 2020). Tortoises shared 15%–31% of all burrows over 2–3 years at three other *G. morafkai* populations in Arizona, and sex‐specific combinations of burrow‐sharing were nonrandom due to females sharing burrows with other females less often than expected (Murray and Klug 1996). In contrast to tortoises at FMR and Sugarloaf, where males and females used similar numbers of burrows, male *G. agassizii* used more burrows per year than females (Harless et al. 2009). In that population males used a greater number of burrows that were also used by other males, presumably due to their use of a greater number of burrows throughout the year. In contrast, females shared more burrows with other females, potentially because favorable nesting sites were limited, causing the females to compete for occupation of burrows (Harless et al. 2009).

### 4.4 Shelter Use Relative to Shelter Type

At FMR, tortoises predominantly used pallets, followed by caliche burrows, more than any other burrow type. However, tortoises used such a low proportion of the available caliche burrows that their visitation rate appeared to avoid caliche due to the much larger number available to choose from than other types (Table 2). Nonetheless, caliche burrows were the only burrow type that was preferred by tortoises based on their observed frequency of occupancy; all other types were occupied in proportion to their availability. Five of the six most‐visited burrows were caliche; two of these were used exclusively during the summer, while the others were used throughout the year, including for hibernation. The relatively high proportion of soil burrows used reflects their scarcity on the landscape; these less permanent structures are typically constructed by tortoises as needed. Unused soil burrows likely were created by other tortoises with overlapping home ranges. Only nine tortoises had access to rock burrows (Appendix F; Riedle et al. 2008), but one of these was among the most used burrows at FMR, exclusively during winter.

Sugarloaf tortoises used more rock burrows and pallets than soil and packrat burrows and used rock and soil burrows in similar proportions to their availability. Like caliche burrows at FMR, Sugarloaf contained so many rock burrows that tortoises appeared to avoid them based on their proportional use. However, occupation rates of all burrow types occurred proportional to availability. Not surprisingly, rock burrows made up all six of the most‐visited burrows, some at specific times of year for particular purposes, such as a preferred nesting site, winter‐specific use, and year‐round use.

Tortoises at FMR clearly preferred caliche burrows even though adequate burrows of other types were available in their home ranges. Rock burrows were so predominant at Sugarloaf that virtually any use of soil or packrat burrows ensured that use essentially was proportional to availability. Sullivan et al. (2014) also found that tortoises used only a small portion of available caliche formations at their study site, and sexes did not differ in the degree of burrow selectivity, as we also found at FMR and Sugarloaf. In contrast, apparent lack of selection for specific overwintering refugium types for Eastern Diamondbacks *Crotalus adamanteus* may be because most of the available refugia provided appropriate microhabitats for overwintering; thus, availability likely had greater influence on refugium use than specific preferences (Murphy et al. 2021).

Caliche burrows were used for hibernation about half of the time at FMR, while rock burrows were used as hibernacula 85% of the time at Sugarloaf. In addition to the two uses of pallets for hibernation by females at Sugarloaf reported here, tortoises used pallets for all or part of hibernation 13 other times between 1996 and 2000 (pers. obs.), 10 times by mature females, twice by immature females, and only twice by males (both near the size of sexual maturity). Female *G. morafkai* at other sites also use superficial refuges to overwinter, with 33% observed in some years by Sullivan and Rubke (2023), while most females and all males hibernated in substantial caliche formations in washes. At a higher elevation site though, Bailey, Schwalbe, and Lowe (1995) observed no use of pallets for hibernation. Use of superficial refuges by females during winter, where not precluded by excessively low temperatures, likely facilitates early emergence and foraging in preparation for egg production the following spring (Averill‐Murray, Christopher, and Henen 2018). In contrast, hibernaculum choices by *G. agassizii* at multiple populations did not clearly affect hibernation temperature or spring emergence differentially for males and females (Nussear et al. 2007).

### 4.5 Importance of Shelters to Desert Tortoise Ecology and Conservation Implications

Mitchell and Powell (2004) developed a model of how animals might choose patches for their home ranges in ways that are optimal with respect to spatially distributed resources. Their model first predicts that animals living in landscapes with slightly to moderately clumped resources should have home ranges highly variable in size, which we found at both FMR and Sugarloaf. High variability would result from colonizing or dominant individuals occupying small, highly efficient home ranges, while new or subordinate individuals needing increasingly larger home ranges to satisfy the same needs. This suggests that tortoises with smaller home ranges might hold more dominant positions in their local social hierarchies than tortoises with larger home ranges, relationships that could be clarified with more focused behavioral study. For example, body size and residency contributed to male dominance in experimental trials with *G. agassizii* (Niblick, Rostal, and Classen 1994), females of both *G. agassizii* and *G. morafkai* segregate burrow use at varying levels (Bulova 1994; Murray and Klug 1996; Averill‐Murray, Fleming, and Riedle 2020; but see Harless et al. 2009), and chemical cues indicative of prior use by other females persuaded female *G. agassizii* to avoid those burrows (Bulova 1997). These observations suggest the possibility of a dominance hierarchy among female desert tortoises or at least a resident advantage that pushes subsequent females further afield in search of a desirable burrow, thereby increasing variation in home‐range sizes.

Next, Mitchell and Powell's model predicts that home‐range overlap should be low and should vary little with resource configuration on the landscape. The mean spatial overlap of home ranges was similar between FMR (0.39) and Sugarloaf (0.34), despite burrow distribution being twice as clumped at FMR. However, even more germane to the question of burrows as a limiting resource, Mitchell and Powell's prediction also was met in that tortoises at both sites shared only two or fewer burrows on average. Even though the rate of sharing was statistically greater at FMR (2.0) than at Sugarloaf (0.9), these represent < 7% of the roughly 30 available burrows within the overlapping portions of their home ranges at both sites.

Mitchell and Powell's model also included predictions for two different strategies for how animals establish their home ranges. The strategy where animals focus on satisfying minimal resource needs (as opposed to maximizing resources within their home ranges) applies best to our study. The model predicts that animals striving to satisfy minimal resource needs in areas with lower resource availability should have larger home ranges because their rate of resource accumulation per unit area decreases while their minimum resource threshold remains the same. As more animals use a given landscape, they sequester the best resources first and reduce the availability to subsequent individuals. This strategy is consistent with our finding that home ranges at FMR with low burrow density were larger than at Sugarloaf with higher burrow density. Home ranges also should have consistent resource levels within populations, which we also saw at FMR and Sugarloaf. Despite differences in burrow density at FMR and Sugarloaf, the number of burrows used by tortoises did not vary between sexes or sites, with tortoises using an average of about 14 burrows within their overall home ranges; they used 8–10 burrows within their core areas (Table 3).

Under the conditions described above, with tortoises needing a threshold number of burrows for their basic life‐history requirements and with those burrows occurring in a patchy distribution across the landscape, as population density increases home ranges should also increase. However, resource depression (i.e., a maximum degree of tolerable burrow sharing between tortoises) sets a limit to the number of home ranges a landscape can support. This limit establishes a carrying capacity for the population (Mitchell and Powell 2004). That burrow availability might set the carrying capacity for *G. morafkai* populations also has previously been suggested based on a strong relationship between burrow abundance and tortoise density across six populations with multi‐year capture–recapture histories (Averill‐Murray, Woodman, and Howland 2002).

Emphasis on protecting areas with high resource abundance—as well as low resource stochasticity—would allow conservation managers to quantify the quality of a habitat based on its resource abundance and stochasticity as well as the amount of space animals would need in the habitat (Mezzini et al. 2023). However, managers also must consider the effects of increasing resource variability and more frequent extreme events to avoid underestimating the space‐use requirements of wildlife. As resource variability increases, the chances of encountering low‐resource patches increase, stochastic environments tend to be less productive, and added movement required to search for the necessary resources increases animals' energetic requirements. Protected areas which were designated based only on the area's mean resource levels may be insufficient in both size and resources in the future. This issue would be particularly acute in resource‐poor, stochastic regions, where animals are forced to endure longer and more unpredictable searches, resulting in more variable space‐use requirements (Mezzini et al. 2023).

To the extent that burrows are the primary limiting resource for populations of *G. morafkai* , one might assume that spatiotemporal variability of burrows is not particularly relevant to conservation management. Those populations with an abundance of rock or caliche burrows may be less affected by changes in extreme temperatures so long as enough burrows of adequate depth remain to buffer increasing temperatures. However, populations more reliant on soil burrows or pallets, like FMR (61% and 55% of all shelters used within total home ranges and core areas, respectively; Table 3), may be more subject to population declines related to increasing temperatures. This would only be exacerbated by human impacts to tortoise habitat below rocky slopes like those at Sugarloaf. For example, as noted above tortoises at FMR selected habitat with greater canopy cover and an absence of cattle activity (Grandmaison, Ingraldi, and Peck 2010). Cattle grazing can negatively affect tortoise habitat by collapsing burrows, trampling vegetation, or contributing to competition for forage (Berry 1978; Nicholson and Humphreys 1981; Avery and Neibergs 1997). Off‐highway vehicle use can have similar effects (Bury and Luckenbach 2002; Averill‐Murray and Allison 2023).

Management practices promoting shelter persistence are valuable to desert tortoises and other wildlife (Murphy et al. 2021). Furthermore, development of low‐lying habitats may have a greater effect on *G. morafkai* populations than initially realized (Howland and Rorabaugh 2002). In addition to direct impacts on tortoises themselves, individuals on the periphery of mountain slopes and bajadas form linkages between disjunct “rock‐pile” populations, including immigrating or emigrating individuals responsible for gene flow between tortoise populations (Edwards et al. 2004; Averill‐Murray and Averill‐Murray 2005). Nonetheless, managers also need to address other threats such as wildfire and activity constraints related to increasing temperatures, among others (Averill‐Murray et al. 2023; Sinervo et al. 2024).

Lastly, the strong ties of *G. morafkai* to their burrows has led some to conclude that *G. morafkai* do not make good candidates for mitigation translocation (Sullivan et al. 2016) even though translocation increasingly has been implemented as a conservation measure for desert tortoises in recent years (e.g., Esque et al. 2010; Scott et al. 2020) and has been suggested as potentially being of even greater importance as a means of assisted migration in the future (Sinervo et al. 2024). However, we point out that this opinion discounts the capacity for learning by turtles (López et al. 2000) as well as the steps that have been taken particularly for *Gopherus* species to ensure that tortoises have the opportunity to learn a new environment. Such steps include making sure that tortoises have adequate space for exploratory movements to find burrows and other resources following release (e.g., by ensuring restricted access to unsuitable habitat or threats on the landscape, such as paved roads and human developments), releasing under appropriate thermal or seasonal conditions, provision of burrows associated with short‐term penning within the release site, etc. (Tuberville et al. 2005; Nussear et al. 2012; Bauder et al. 2014; Paloma Ramos, Colima, and Arana 2018; Dickson et al. 2019). Nevertheless, we agree that mitigation translocations should be minimized in favor of in situ habitat protection as much as possible, translocations should be assessed from an overarching conservation perspective, release sites require thorough evaluation, and translocations should follow scientific best practices for implementation, monitoring, and reporting (Germano et al. 2015; Sullivan, Nowak, and Kwiatkowski 2015; Mack and Berry 2023).

## Author Contributions

**Roy C. Averill‐Murray:** conceptualization (lead), data curation (lead), formal analysis (lead), funding acquisition (lead), investigation (equal), methodology (lead), project administration (lead), supervision (lead), visualization (lead), writing – original draft (lead), writing – review and editing (equal). **J. Daren Riedle:** conceptualization (supporting), data curation (supporting), investigation (equal), resources (lead), writing – review and editing (equal).

## Conflicts of Interest

The authors declare no conflicts of interest.

## Data Availability

Locational data that support the findings of this study (i.e., generation of home ranges) are not publicly available due to the sensitive and protected status of *G. morafkai* . Inquiries about these data should be made to the Arizona Game and Fish Department. Non‐locational data that support the findings of this study are openly available in FigShare within the project “Shelter Distribution and Type Affect Space Use of Sonoran Desert Tortoises ( *Gopherus morafkai* )” at https://figshare.com/account/home#/projects/222894: https://doi.org/10.6084/m9.figshare.27161955.v1, https://doi.org/10.6084/m9.figshare.27161985.v1, https://doi.org/10.6084/m9.figshare.27162006.v1, https://doi.org/10.6084/m9.figshare.27162024.v1, https://doi.org/10.6084/m9.figshare.27162036.v1, https://doi.org/10.6084/m9.figshare.27162048.v1, and https://doi.org/10.6084/m9.figshare.27162108.v1.

## Appendix A

Hypotheses, final model structure (in italics) after modifications indicated by model selection and diagnostics, and results and conclusions from analyses of Sonoran Desert Tortoise burrow and space use at Sugarloaf Mountain (Sugar) and Florence Military Reservation (FMR), Arizona. Unless otherwise indicated, beta estimates are given for all continuous variables and for factors lacking clear differences, and pairwise comparisons are given for factors showing clear differences. 95% CIs are given in parentheses. Clear statistical differences are indicated with an asterisk or with different alphabetic superscripts for multiple pairwise comparisons. GLS, generalized least squares regression; GLMM, generalized linear mixed model; HR, home range. R packages used are given in footnotes.

**Table:**

Contrast/coefficient	Conclusion
1. *H*_0_: Difference in wAKDE_C_ HR between females (F) and males (M) = 0; difference between sites = 0; regression slope of number of burrows = 0.	
*Mixed‐effects meta‐analysis* ^1^ *: AKDE ~ sex + site + ln*(*burrows*)	
${\hat{\beta }}_{\ln\left(\text{burrows}\right)}$ = 3.06 (−0.14 to 6.25)	*HR size increased with more burrows (significant effect in permutation test).
F–M = −4.4 (−8.2 to −0.7)	*HRs smaller for F than for M.
FMR – Sugar = 6.4 (3.2–9.7)	*HRs larger at FMR than at Sugarloaf.
2. *H*_0_: Ratio of burrow density in wAKDE_C_ HR between F and M = 1; ratio between sites = 1.	
*GLS* ^2^ *: ln*(*burrow density*) *~ sex + site*	
${\hat{\beta }}_{\mathrm{sex}}$ = −0.23 (−0.61–0.14)	No clear difference in burrow density between F and M home ranges.
FMR:Sugar = 0.33 (0.23–0.47)	*FMR home ranges contain ~33% the burrow density as Sugarloaf home ranges.
3. *H*_0_: Difference in wAKDE_C_ 50% core area between F and M = 0; difference between sites = 0; regression slope of number of burrows = 0.	
*Mixed‐effects meta‐analysis* ^1^ *: Core ~ sex + site + sqrt*(*burrows*)	
${\hat{\beta }}_{\text{sqrt}\left(\text{burrows}\right)}$ = 0.37 (−0.03 to 0.77)	*Core size increased with more burrows (significant effect in permutation test).
F–M = −1.2 (−2.5 to 0.1)	*Core areas smaller for F than for M.
FMR–Sugar = 1.8 (0.5–3.0)	*Core areas larger at FMR than at Sugarloaf.
4. *H*_0_: Difference in wAKDE_C_ 50%–95% noncore area between F and M = 0; difference between sites = 0; regression slope of number of burrows = 0.	
*Mixed‐effects meta‐analysis* ^1^ *: Noncore ~ site* × *ln*(*burrows*) *+ sex*	
${\hat{\beta }}_{\text{site}*\text{burrows}}$ = −25.6 (−37.7 to −13.6)	*Noncore size increased more rapidly with number of burrows at FMR than at Sugarloaf.
FMR–Sugar = 10.7 (6.7–14.6)	*For a given number of burrows, FMR noncore areas larger than at Sugarloaf.
${\hat{\beta }}_{\mathrm{sex}}$ = −1.0 (−10.0 to 7.9)	Size of noncore areas did not clearly differ between sexes.
5. *H*_0_: Difference in burrow density between core and noncore areas = 0 at both sites and between both sexes.	
*Repeated‐measures ANOVA* ^3^ *: sqrt*(*burrow density*) *~ site* × *sex* × *HR portion*	
Core–Noncore (FMR) = 1.0 (0.4–1.6)	*Burrow density is greater in core areas than noncore areas at FMR.
Core–Noncore (Sugar) = 0.7 (0.2–1.3)	*Burrow density is greater in core areas than noncore areas at Sugarloaf.
Core–Noncore (F) = 0.9 (0.4–1.5)	*Burrow density is greater in core areas than noncore areas for F.
Core–Noncore (M) = 0.6 (−0.3–1.6)	Burrow density is not clearly different between core and noncore areas for M.
6. *H*_0_: Regression slope of intensity of core area use (ICU) versus number of core area burrows, site, and sex = 0.	
*GLM* ^4^ *: ICU ~ sex + site + # core area burrows, family = Gamma* (*link = log*)	
${\hat{\beta }}_{\mathrm{sex}}$ = 0.01 (−0.15 to 0.17)	Intensity of core area use did not clearly differ between sexes.
${\hat{\beta }}_{\text{site}}$ = 0.09 (−0.06 to 0.24)	Intensity of core area use did not clearly differ between sites.
${\hat{\beta }}_{\#\text{burrows}}$ = −0.006 (−0.010 to −0.002)	*Intensity of core area use decreases with the number of burrows in the core area.
7. *H*_0_: Difference in burrows used in 95% wAKDE_C_ HR between F and M = 0; difference between FMR and Sugarloaf = 0; regression slope of number of available burrows = 0.	
*GLS* ^2^ *: # used burrows ~ sex + site + # available burrows*	
$\;{\hat{\beta }}_{\mathrm{sex}}$ = 0.7 (−3.5 to 4.9)	Numbers of burrows used in the total HR did not clearly differ between sexes.
${\hat{\beta }}_{\text{site}}$ = −1.2 (−5.1 to 2.7)	Numbers of burrows used in the total HR did not clearly differ between sites.
${\hat{\beta }}_{\#\text{available burrows}}$ = 0.02 (−0.02 to 0.06)	Numbers of burrows used in the total HR is not clearly related to the number available.
8. *H*_0_: Difference in burrows used in core areas between F and M = 0; difference between FMR and Sugarloaf = 0; regression slope of number of available burrows = 0.	
*GLS* ^2^ *: # used burrows ~ sex + site + # available burrows*	
${\hat{\beta }}_{\mathrm{sex}}$ = −1.1 (−3.9–1.7)	Numbers of burrows used in core areas did not clearly differ between sexes.
${\hat{\beta }}_{\text{site}}$ = −2.2 (−4.8–0.3)	Numbers of burrows used in core areas did not clearly differ between sites.
${\hat{\beta }}_{\#\text{available burrows}}$ = 0.13 (0.05–0.21)	*Numbers of burrows used in core areas increased with the number available.
9. *H*_0_: Difference in burrows used in noncore areas between F and M = 0; difference between FMR and Sugarloaf = 0; regression slope of number of available burrows = 0.	
*GLS* ^2^ *: # used burrows ~ sex + site + # available burrows*	
${\hat{\beta }}_{\mathrm{sex}}$ = −1.2 (−4.4–1.9)	Numbers of burrows used in noncore areas did not clearly differ between sexes.
${\hat{\beta }}_{\text{site}}$ = −2.8 (−5.7–0.1)	Numbers of burrows used in noncore areas did not clearly differ between sites.
${\hat{\beta }}_{\#\text{available burrows}}$ = 0.03 (−0.02–0.07)	Numbers of burrows used in noncore areas is not clearly related to the number available.
10. *H*_0_: Difference in mean number of visits to individual burrows between F and M = 0; difference between FMR and Sugarloaf = 0.	
*GLS* ^2^ *: Mean visits ~ sex* × *site, weights = Identity*(*sex*)	
FMR F–M = 0.03 (−0.20 to 0.25)	Visits to individual burrows did not clearly differ between sexes at FMR.
Sugar F–M = 0.37 (0.16–0.57)	*Females visited individual burrows more often than did males at Sugarloaf.

11. *H*_0_: Difference in time spent within burrows between F and M = 0; difference between FMR and Sugarloaf = 0; regression slope of entrance day = 0.		
*GLMM* ^4^: *Duration ~ sex + site * poly*(*entrance_day, 4*) + (*1|shelter*) + (*1|ID*), *family = Gamma* (*link = log*)		
F:M = 1.22 (1.01–1.47)		*Females spent ~22% more time in burrows than did males.
Site*${\hat{\beta }}_{\mathrm{day}1}$ = 2.3 (−4.0 to 8.5)	Site*${\hat{\beta }}_{\mathrm{day}3}$ = 6.3 (0.2–12.3)	*Tortoises spent relatively little time burrows that they entered during spring, but this decreased even further for burrows entered during summer. Time spent in burrows increased as tortoises entered winter hibernation, even more so at FMR.
Site*${\hat{\beta }}_{\mathrm{day}2}$ = −4.1 (−11.7 to 3.5)	Site*${\hat{\beta }}_{\mathrm{day}4}$ = 6.6 (1.7–11.5)	
12. *H*_0_: Difference in number of shared burrows between sex dyads = 0; difference between FMR and Sugarloaf = 0.		
*GLM* ^4^ *: Shared burrows ~* (*site * log*_*10*_(*fixes*)) *+ dyad + log*_*10*_(*HR overlap*), *family = Poisson, offset = log*_*10*_(*available burrows*)		
${\hat{\beta }}_{\text{overlap}}$ = 3.09 (2.35–3.84)	*Number of shared burrows increased with the proportion of HR overlap.	
${\hat{\beta }}_{\text{site}*\text{fixes}}$ = 2.97 (0.92–5.01)	*Number of shared burrows at Sugarloaf increased more quickly with number of fixes/pair than at FMR.	
${\hat{\beta }}_{\text{fixes}}$ = 1.88 (0.53–3.23)	*Number of shared burrows increased with number of fixes/pair.	
FMR:Sugar = 1.72 (1.24–2.37)	*Pairs of tortoises at FMR shared 72% more burrows on average than pairs at Sugarloaf.	
F–F:F–M = 0.67 (0.46–0.97)	*Pairs of female tortoises shared 33% fewer burrows than female–male pairs.	
F–F:M–M = 0.60 (0.33–1.09)	Rate of burrows sharing did not clearly differ between pairs of females and pairs of males.	
F–M:M–M = 0.89 (0.52–1.52)	Rate of burrows sharing did not clearly differ between female–male pairs and pairs of males.	
13. *H*_0_: Difference at FMR in numbers of shelters used between shelter types = 0; difference between F and M = 0.		
*LMM* ^2^ *: Total shelters used ~ sex* × *shelter type* (*random = ~1|ID, weights = Identity*(*sex*))		
${\hat{\beta }}_{\mathrm{sex}}$ = 0.11 (−1.65 to 1.88)		The total number of shelters used at FMR did not clearly differ by sex.
Caliche = 6.4 (4.2–8.5)^b^	Rock = 2.4 (0.6–4.1)^c^	*Tortoises at FMR used more pallets than all other shelter types and more unique caliche burrows than rock or soil burrows. Marginal means with different superscripts are significant at α = 0.05.
Pallet = 16.1 (10.6–21.7)^a^	Soil = 2.8 (1.4–4.2)^c^	
Packrat = 3.4 (1.4–5.4)^bc^		
14. *H*_0_: Difference at FMR in the proportion of burrows used by type = 0; difference between F and M = 0.		
*GLMM* ^4^ *: Proportion of type ~ burrow type* + *sex +* (*1|ID*), *family = binomial*		
${\hat{\beta }}_{\mathrm{sex}}$ = 0.10 (−0.58 to 0.78)		The proportional use of different burrow types at FMR did not clearly differ by sex.
Caliche = 0.17 (0.13–0.23)^a^	Rock = 0.30 (0.21–0.42)^b^	*Tortoises at FMR used a lower proportion of caliche burrows than other types and a higher proportion of soil burrows than rock burrows. Marginal means with different superscripts are significant at α = 0.05.
Packrat = 0.35 (0.26–0.45)^b,c^	Soil = 0.50 (0.38–0.62)^c^	

15. *H*_0_: Tortoises at FMR visit each burrow type in proportion to its availability. f = proportion of tortoises which use that type in a greater proportion than its availability. Values on the diagonal are *p*‐values for each partial hypothesis of proportional burrow use; the non‐diagonal value is the *p*‐value for paired comparisons among caliche and packrat burrow types^5^						
**Burrow type**	***f***	**Soil**	**Rock**	**Packrat**	**Caliche**	**Use**
Soil	0.5294	1.000				Proportional
Rock	0.2222		0.1797			Proportional
Packrat	0.2000			0.0352	0.3229	Avoided
Caliche	0.0556				0.0001	Avoided
16. *H*_0_: Tortoises at FMR occupy each burrow type in proportion to its availability. Values as above^5^						
**Burrow type**	***f***	**Soil**	**Rock**	**Packrat**	**Caliche**	**Use**
Caliche	0.8333	0.0075				Preferred
Soil	0.5882		0.6291			Proportional
Rock	0.4444			1.0000		Proportional
Packrat	0.4286				0.7905	Proportional

17. *H*_0_: Difference at Sugarloaf in numbers of shelters used between shelter types = 0; difference between F and M = 0.		
*LMM* ^2^ *: Total shelters used ~ shelter type* × *sex* (*random = ~1|ID, weights = Identity*(*sex*))		
$\;{\hat{\beta }}_{\mathrm{sex}}$ = 0.07 (−0.28–0.42)		The total number of shelters used at Sugarloaf did not clearly differ by sex.
Pallet = 13.0 (9.1–16.8)^a^	Rock = 11.6 (9.1–14.1)^a^	*Tortoises at Sugarloaf used more pallets and rock burrows than packrat and soil burrows; tortoises used more soil than packrat burrows. Marginal means with different superscripts are significant at α = 0.05.
Packrat = 0.1 (−0.03 to 0.3)^b^	Soil = 2.1 (1.4–2.9)^c^	
18. *H*_0_: Difference at Sugarloaf in the proportion of burrows used by type = 0; difference between F and M = 0.		
*GLMM* ^4^ *: Proportion of type ~ shelter type* + *sex +* (*1|ID*), *family = binomial*		
${\hat{\beta }}_{\mathrm{sex}}$ = −0.57 (−1.49–0.35)		Proportional use of different burrow types at Sugarloaf did not clearly differ by sex.
Packrat = 0.26 (0.07–0.61)	Soil = 0.19 (0.12–0.29)	There were no clear deviations from proportional use of each burrow type at Sugarloaf (values are marginal means).
Rock = 0.16 (0.11–0.24)		

19. *H*_0_: Tortoises at Sugarloaf visit each burrow type in proportion to its availability (packrat burrows excluded due to lack of visits). f = proportion of tortoises which uses that type in a greater proportion than its availability. Values on the diagonal are *p*‐values for each partial hypothesis of proportional burrow use; the non‐diagonal value is the *p*‐values for paired comparisons among rock and soil burrow types^5^				
**Burrow type**	***f***	**Soil**	**Rock**	**Use**
Soil	0.2609	0.0347	0.9995	Avoided
Rock	0.2500		0.0227	Avoided

20. *H*_0_: Tortoises at Sugarloaf occupy each burrow type in proportion to its availability. Values as above^5^					
**Burrow type**	***f***	**Rock**	**Soil**	**Packrat**	**Use**
Rock	0.5833	0.5413			Proportional
Soil	0.3333		0.1516		Proportional
Packrat	0.2857			0.4531	Proportional

^1^ *metafor* (Viechtbauer 2010).

^2^ *nlme* (Piniero et al. 2023).

^3^ *rstatix* (Kassambara 2023).

^4^ *glmmTMB* (Brooks et al. 2017).

^5^ *phuassess* (Pisani 2016).

## Appendix B

*Gopherus morafkai* home range estimates at the Florence Military Reservation, Arizona, 2000–2003. $\hat{N}$_area_ = effective sample size; AKDE = area‐corrected, optimally weighted, autocorrelated kernel density estimation home range; PA = proportion of the 95% AKDE contained by the 50% core area; ICU = intensity of core area use; MCP = minimum convex polygon estimates from Riedle et al. (2008).

**Table:**

Sex/ID	No. fixes	No. years	Mean sample interval (d)	Top model^a^	$\hat{N}$_area_	95% AKDE (ha), 95% CI	Var	50% Core AKDE (ha), 95% CI	PA	ICU	50%–95% AKDE (ha)	MCP (ha)
M#400	127	4	7.0	1	126	16.3 13.5–19.2	2.11	3.1 2.6–3.7	0.19	2.63	13.2	23.5
M#403	144	4	6.8	2	67.1	102.5 79.4–128.4	156.58	18.8 14.6–23.5	0.18	2.73	83.7	93.4
M#406	163	4	5.5	2	43.4	11.7 8.5–15.4	3.15	2.5 1.8–3.2	0.21	2.34	9.2	9.7
M#411	83	4	5.5	2	34.7	14.9 10.4–20.3	6.40	3.7 2.6–5.0	0.25	2.01	11.2	9.0
M#413	107	3	4.4	2	52.5	36.0 26.9–46.4	24.69	6.1 4.6–7.9	0.17	2.95	29.9	43.1
M#414	79	2	4.3	1	78	20.2 15.9–24.9	5.23	3.1 2.5–3.8	0.15	3.26	17.1	29.1
M#419	149	3	4.5	2	67.4	27.6 21.4–34.5	11.30	7.2 5.6–9.0	0.26	1.92	20.4	25.7
F#404	202	4	5.2	2	67.7	21.6 16.8–27.1	6.89	4.4 3.4–5.5	0.20	2.45	17.2	28.1
F#405	158	4	5.5	3	157	6.3 5.4–7.4	0.25	1.5 1.3–1.8	0.24	2.10	4.8	5.1
F#408	28	2	10.7	3	27	10.5 6.9–14.8	4.08	2.7 1.8–3.8	0.26	1.94	7.8	5.0
F#410	128	4	6.4	4	127.0	46.8 39.0–55.3	17.25	7.9 6.6–9.3	0.17	2.96	38.9	46.2
F#412	160	3	5.0	1	159	15.4 13.1–17.9	1.49	3.2 2.8–3.8	0.21	2.41	12.2	17.4
F#420	155	3	4.0	1	154	10.7 9.1–12.5	0.74	2.0 1.7–2.3	0.19	2.68	8.7	11.2
F#421	89	3	5.5	2	28.3	7.6 5.0–10.6	2.04	2.2 1.5–3.1	0.29	1.73	5.4	3.1
F#430	83	2	4.5	5	37.1	16.7 11.7–22.4	7.52	4.3 3.1–5.8	0.26	1.94	12.4	10.0
F#501	30	2	9.2	2	9.7	135.8 64.3–233.6	1901.20	36.8 17.4–63.3	0.27	1.85	99.0	52.8
F#502	145	4	5.8	2	61.2	8.8 6.7–11.1	1.27	1.7 1.3–2.1	0.19	2.59	7.1	12.0
F#503	59	2	5.7	5	33.1	9.7 6.7–13.3	2.84	2.8 1.9–3.8	0.29	1.73	6.9	5.5

^a^ Models: 1 = IID anisotropic, 2 = OU anisotropic, 3 = IID, 4 = OUF anisotropic, and 5 = OUF.

## Appendix C

*Gopherus morafkai* home range estimates at Sugarloaf Mountain, Arizona, 2000–2003. $\hat{N}$_area_ = effective sample size; AKDE = area‐corrected, optimally weighted, autocorrelated kernel density estimation home range; PA = proportion of the 95% AKDE contained by the 50% core area; ICU = intensity of core area use; Full 95% AKDE = cumulative AKDE estimates from 1996 through 2005 reported by Averill‐Murray, Fleming, and Riedle (2020).

**Table:**

Sex/ID	No. fixes	No. years	Mean sample interval (d)	Top model^a^	$\hat{N}$_area_	95% AKDE (ha), 95% CI	Var	50% Core AKDE (ha), 95% CI	PA	ICU	50%–95% AKDE (ha)	Full 95% AKDE (ha), 95% CI	Full top model^a^
M#9	97	3	7.5	1	52.7	9.1 6.8–11.7	1.57	2.1 1.6–2.7	0.23	2.17	7.0	10.5 8.5–12.8	1
M#20	62	3	7.6	1	39.4	12.0 8.5–16.0	3.65	2.4 1.7–3.2	0.20	2.50	9.6	16.8 12.6–21.6	1
M#48	68	3	7.1	1	46.5	13.7 10.0–17.9	4.04	3.3 2.4–4.3	0.24	2.08	10.4	12.3 9.6–15.2	1
M#59	66	3	9.7	1	45.6	14.7 10.8–19.3	4.74	3.1 2.2–4.0	0.21	2.37	11.6	14.7 11.3–18.6	1
M#60	67	3	9.1	1	41.1	9.0 6.5–11.9	1.97	2.0 1.5–2.7	0.22	2.25	7.0	8.6 6.2–11.2	1
F#1	145	4	7.7	1	78.0	3.6 2.8–4.4	0.17	0.8 0.7–1.0	0.22	2.25	2.8	3.4 2.9–4.0	1
F#3	154	4	7.2	1	79.5	5.9 4.7–7.3	0.44	1.0 0.8–1.2	0.17	2.95	4.9	6.2 5.3–7.1	1
F#17	154	4	7.2	1	68.8	3.2 2.5–4.0	0.15	0.8 0.6–0.9	0.25	2.00	2.4	5.6 4.5–6.8	1
F#29	129	4	7.3	2	103.6	3.7 3.0–4.4	0.13	0.5 0.4–0.6	0.14	3.70	3.2	3.6 3.1–4.2	1
F#46	109	3	7.2	1	51.6	6.6 4.9–8.5	0.84	1.7 1.3–2.2	0.26	1.94	4.9	7.6 6.4–8.8	1
F#57	60	3	9.9	3	57.5	1.2 0.9–1.6	0.03	0.1 0.08–0.13	0.08	6.00	1.1	4.5 3.7–5.4	1
F#58	133	4	7.3	2	115.5	3.9 3.2–4.6	0.13	0.6 0.5–0.7	0.15	3.25	3.3	4.9 4.3–5.6	1
F#63	131	4	7.0	1	54.0	10.0 7.5–12.8	1.85	2.5 1.9–3.2	0.25	2.00	7.5	10.5 8.9–12.2	1
F#65	50	2	7.0	2	37.8	7.6 5.4–10.3	1.53	1.5 1.1–2.1	0.20	2.53	6.1	8.9 7.3–10.5	1
F#66	148	4	7.0	1	62.2	7.2 5.5–9.1	0.83	1.3 1.0–1.7	0.18	2.77	5.9	6.1 5.0–7.2	1
F#67	29	1	7.0	1	16.8	12.1 7.0–18.6	8.71	2.5 1.4–3.8	0.21	2.42	9.6	15.1 10.8–20.2	1
F#68	157	4	7.1	1	88.2	2.1 1.7–2.6	0.05	0.3 0.22–0.33	0.14	3.50	1.8	2.5 2.2–2.7	4
F#69	62	2	7.2	1	9.6	20.9 9.9–36.0	45.50	5.2 2.4–8.9	0.25	2.01	15.7	19.6 11.7–29.6	1
F#71	25	1	7.1	2	15.5	6.5 3.7–10.1	2.73	1.5 0.8–2.3	0.23	2.17	5.0	11.0 8.1–14.4	1
F#72	158	4	7.1	1	102.9	3.9 3.1–4.6	0.15	1.1 0.9–1.4	0.28	1.77	2.8	4.1 3.5–4.8	1
F#80	31	1	6.8	1	15.4	11.9 5.4–20.9	9.20	3.3 1.5–5.8	0.28	1.80	8.6	11.5 7.7–16.1	1
F#81	25	1	6.7	1	10.7	18.7 9.3–31.5	32.68	3.2 1.6–5.5	0.17	2.92	15.5	11.2 8.4–14.5	1
F#86	149	4	7.2	1	57.5	6.2 4.7–7.8	0.67	1.3 1.0–1.7	0.21	2.38	4.9	6.3 5.2–7.4	1
F#625	28	1	7.9	1^b^	10.8	12.3 6.1–20.6	14.01	2.5 1.2–4.2	0.20	2.46	9.8	7.8 5.3–10.8	1

^a^ Models: 1 = OU anisotropic, 2 = OUf anisotropic, 3 = OUf, and 4 = IID; isotropy not reported by Averill‐Murray, Fleming, and Riedle (2020).

^b^ Bootstrapped due to estimated 1.6% bias in initial analysis.

## Appendix D

Numbers of shelters within *Gopherus morafkai* home ranges, core areas, and noncore areas at Florence Military Reservation, 2000–2003. wAKDE_C_ = area‐corrected, optimally weighted, autocorrelated kernel density estimation home range; Rat = packrat.

**Table:**

Sex/ID	95% wAKDE_C_					50% Core Areas						50%–95% Noncore Areas					
	Total	Caliche	Rock	Soil	Rat	Total	Density (ha^−1^)	Caliche	Rock	Soil	Rat	Total	Density (ha^−1^)	Caliche	Rock	Soil	Rat
M#400	62	42	0	3	17	34	11.0	28	0	0	6	28	2.1	14	0	3	11
M#403	115	83	0	14	18	24	1.3	18	0	4	2	91	1.1	65	0	10	16
M#406	65	56	0	3	6	18	7.2	18	0	0	0	47	5.1	38	0	3	6
M#411	42	38	0	3	1	21	5.7	17	0	3	1	21	1.9	21	0	0	0
M#413	40	29	2	4	5	25	4.1	23	0	0	2	15	0.5	6	2	4	3
M#414	49	21	26	1	1	6	1.9	1	4	0	1	43	2.5	20	22	1	0
M#419	107	47	28	14	18	47	6.5	5	16	11	15	60	2.9	42	12	3	3
F#404	53	34	0	3	16	21	4.8	19	0	1	1	32	1.9	15	0	2	15
F#405	41	24	0	2	15	23	15.3	13	0	0	10	18	3.8	11	0	2	5
F#408	32	21	9	2	0	19	7.0	16	2	1	0	13	1.7	5	7	1	0
F#410	87	65	0	8	14	32	4.1	30	0	1	1	55	1.4	35	0	7	13
F#412	41	33	0	8	0	26	8.1	20	0	6	0	15	1.2	13	0	2	0
F#420	66	25	12	13	16	29	14.5	0	7	7	15	37	4.3	25	5	6	1
F#421	42	21	21	0	0	29	13.2	15	14	0	0	13	2.4	6	7	0	0
F#430	56	21	28	3	4	35	8.1	16	19	0	0	21	1.7	5	9	3	4
F#501	162	90	31	19	22	41	1.1	33	2	4	2	121	1.2	57	29	15	20
F#502	42	25	0	3	14	11	6.5	0	0	2	9	31	4.4	25	0	1	5
F#503	40	5	7	12	16	16	5.7	0	3	8	5	24	3.5	5	4	4	11

## Appendix E

Numbers of shelters within *Gopherus morafkai* home ranges, core areas, and noncore areas at Sugarloaf, 2000–2003. wAKDE_C_ = area‐corrected, optimally weighted, autocorrelated kernel density estimation home range; Rat = packrat.

**Table:**

Sex/ID	95% wAKDE_C_				50% Core Areas					50%–95% Noncore Areas				
	Total	Rock	Soil	Rat	Total	Density (ha^−1^)	Rock	Soil	Rat	Total	Density (ha^−1^)	Rock	Soil	Rat
M#9	106	94	12	0	53	25.2	49	4	0	53	7.6	45	8	0
M#20	63	46	15	2	23	9.6	15	7	1	40	4.2	31	8	1
M#48	134	118	16	0	20	6.1	18	2	0	114	11.0	100	14	0
M#59	98	87	11	0	5	1.6	5	0	0	93	8.0	82	11	0
M#60	96	84	12	0	38	19.0	36	2	0	58	8.3	48	10	0
F#1	70	66	4	0	25	31.3	25	0	0	45	16.1	41	4	0
F#3	88	80	8	0	17	17.0	14	3	0	71	14.5	66	5	0
F#17	42	33	9	0	19	23.8	11	8	0	23	9.6	22	1	0
F#29	40	37	2	1	6	12.0	6	0	0	34	10.6	31	2	1
F#46	123	111	11	1	36	21.2	33	3	0	87	17.8	78	8	1
F#57	33	32	1	0	3	30.0	3	0	0	30	27.3	29	1	0
F#58	74	72	2	0	20	33.3	20	0	0	54	16.4	52	2	0
F#63	55	38	17	0	13	5.2	9	4	0	42	5.6	29	13	0
F#65	91	82	9	0	31	20.7	29	2	0	60	9.8	53	7	0
F#66	40	33	7	0	11	8.5	10	1	0	29	4.9	23	6	0
F#67	96	88	7	1	28	11.2	24	4	0	68	7.1	64	3	1
F#68	13	10	3	0	1	3.3	1	0	0	12	6.7	9	3	0
F#69	272	250	22	0	98	18.8	84	14	0	174	11.1	166	8	0
F#71	29	15	13	1	9	6.0	6	3	0	20	4.0	9	10	1
F#72	61	50	11	0	30	27.3	26	4	0	31	11.1	24	7	0
F#80	36	21	14	1	19	5.8	12	7	0	17	2.0	9	7	1
F#81	126	117	9	0	25	7.8	24	1	0	101	6.5	93	8	0
F#86	40	26	12	2	17	13.1	10	7	0	23	4.7	16	5	2
F#625	141	128	13	0	47	18.8	47	0	0	94	9.6	81	13	0

## Appendix F

Numbers of shelters used by type by *Gopherus morafkai* at Florence Military Reservation, 2000–2003. wAKDE_C_ = area‐corrected, optimally weighted, autocorrelated kernel density estimation home range; Rat = packrat.

**Table:**

Sex/ID	95% wAKDE_C_					50% Core areas					50%–95% Noncore areas				
	Caliche	Rock	Soil	Rat	Pallet	Caliche	Rock	Soil	Rat	Pallet	Caliche	Rock	Soil	Rat	Pallet
M#400	12	0	1	9	36	9	0	0	4	7	3	0	1	5	29
M#403	13	0	2	2	22	6	0	1	2	14	7	0	1	0	8
M#406	15	0	0	0	5	7	0	0	0	4	8	0	0	0	1
M#411	11	0	1	1	5	8	0	1	1	4	3	0	0	0	1
M#413^a^	3	0	4	5	10	2	0	0	2	5	1	0	4	3	5
M#414	2	6	0	1	13	0	1	0	1	5	2	5	0	0	8
M#419	4	8	7	8	19	1	7	5	7	10	3	1	2	1	9
F#404	8	0	0	2	42	7	0	0	1	23	1	0	0	1	19
F#405	6	0	2	10	16	4	0	0	6	7	2	0	2	4	9
F#408	4	1	2	0	1	4	1	1	0	0	0	0	1	0	1
F#410	12	0	2	2	22	11	0	0	1	8	1	0	2	1	14
F#412	4	0	8	0	21	4	0	6	0	6	0	0	2	0	15
F#420	3	5	7	9	19	0	4	6	8	12	3	1	1	1	7
F#421	6	9	0	0	9	4	7	0	0	7	2	2	0	0	2
F#430	3	9	1	0	15	3	9	0	0	8	0	0	1	0	7
F#501	2	0	3	2	3	2	0	3	2	3	0	0	0	0	0
F#502	5	0	3	10	25	0	0	2	9	14	5	0	1	1	11
F#503	1	4	7	0	7	0	2	7	0	4	1	2	0	0	3

^a^ Riedle et al. (2008) reported 0% burrow use.

## Appendix G

Numbers of shelters used by type by *Gopherus morafkai* at Sugarloaf Mountain, 2000–2003. wAKDE_C_ = area‐corrected, optimally weighted, autocorrelated kernel density estimation home range; Rat = packrat.

**Table:**

Sex/ID	95% wAKDE_C_				50% Core areas				50%–95% Noncore areas			
	Rock	Soil	Rat	Pallet	Rock	Soil	Rat	Pallet	Rock	Soil	Rat	Pallet
M#9	14	1	0	8	11	0	0	3	3	1	0	5
M#20	11	2	1	31	6	1	1	23	5	1	0	8
M#48	15	0	0	6	7	0	0	2	8	0	0	4
M#59	8	3	0	14	4	0	0	8	4	3	0	6
M#60	9	2	0	10	5	1	0	5	4	1	0	5
F#1	23	1	0	24	13	0	0	12	10	1	0	12
F#3	21	4	0	11	8	3	0	3	13	1	0	8
F#17	14	5	0	11	7	5	0	5	7	0	0	6
F#29	14	0	1	9	4	0	0	3	10	0	1	6
F#46	22	2	0	5	14	0	0	1	8	2	0	4
F#57	7	0	0	1	2	0	0	0	5	0	0	1
F#58	16	1	0	6	8	0	0	1	8	1	0	5
F#63	9	5	0	9	6	2	0	4	3	3	0	5
F#65	5	1	0	9	3	1	0	7	2	0	0	2
F#66	11	2	0	23	7	1	0	11	4	1	0	12
F#67	7	0	0	10	4	0	0	6	3	0	0	4
F#68	8	3	0	24	1	0	0	10	7	3	0	14
F#69	17	2	0	15	13	2	0	10	4	0	0	5
F#71	2	4	0	4	2	2	0	1	0	2	0	3
F#72	18	4	0	18	15	3	0	10	3	1	0	8
F#80	2	4	1	6	2	3	0	4	0	1	1	2
F#81	10	0	0	8	8	0	0	3	2	0	0	5
F#86	9	5	0	38	3	5	0	30	6	0	0	8
F#625	5	0	0	11	4	0	0	9	1	0	0	2
